# Supplementary material for: The giant diploid faba genome unlocks variation in a global protein crop
Source: Nature. 2023 Mar 8;615(7953):652–9. doi: 10.1038/s41586-023-05791-5 (PMC10033403; doi:10.1038/s41586-023-05791-5)
Supplement: Supplementary file 1 — This file contains Supplementary Figs. 1–17, Supplementary Tables 3, 4, 6, 13 and Supplementary Note (Pretzel instructions). The file supports assembly and structural annotation quality control, gene family and genome duplication analysis, transposable element and DNA methylation analysis, GWAS and gene expression profiling quality control, further information supporting hilum colour and seed size candidate loci. Supplementary Note includes instructions for accessing integrated faba bean datasets using Pretzel. [file 41586_2023_5791_MOESM1_ESM.pdf]

nature portfolio

https://doi.org/10.1038/s41586-023-05791-5

Supplementary information

The giant diploid faba genome unlocks variation in a global protein crop

In the format provided by the authors and unedited

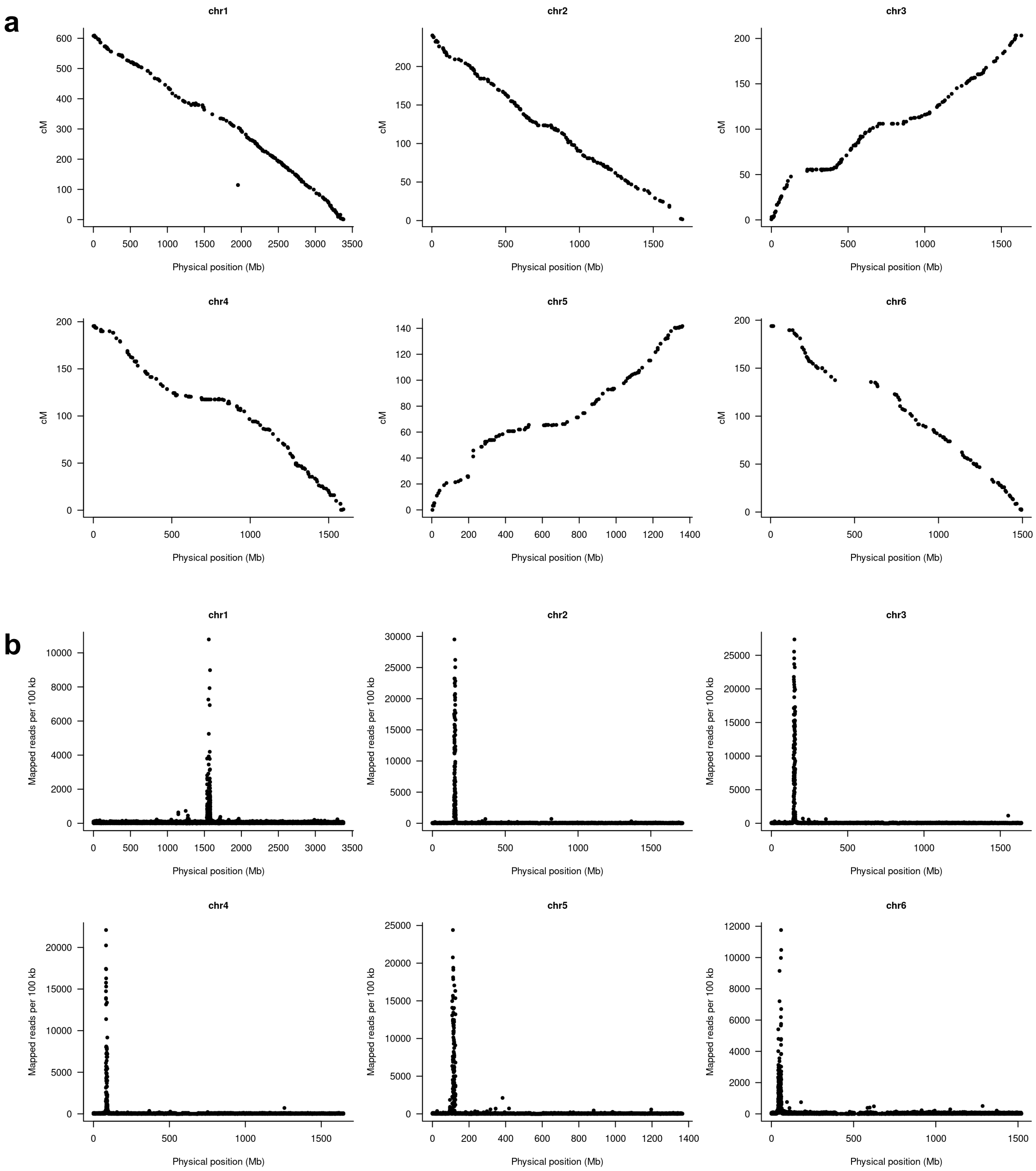

Supplementary Figure 1. Collinearity of physical and genetic maps and ChIP-seq localisation of centromeres. **a,** Collinearity of physical and genetic maps. The antidiagonal alignments in chr1, chr2, chr4, and chr6 were a result of the arbitrary orientation of linkage groups in prior genetic maps. **b,** chromatin immunoprecipitation sequencing (ChIP-seq) localization of centromeres.

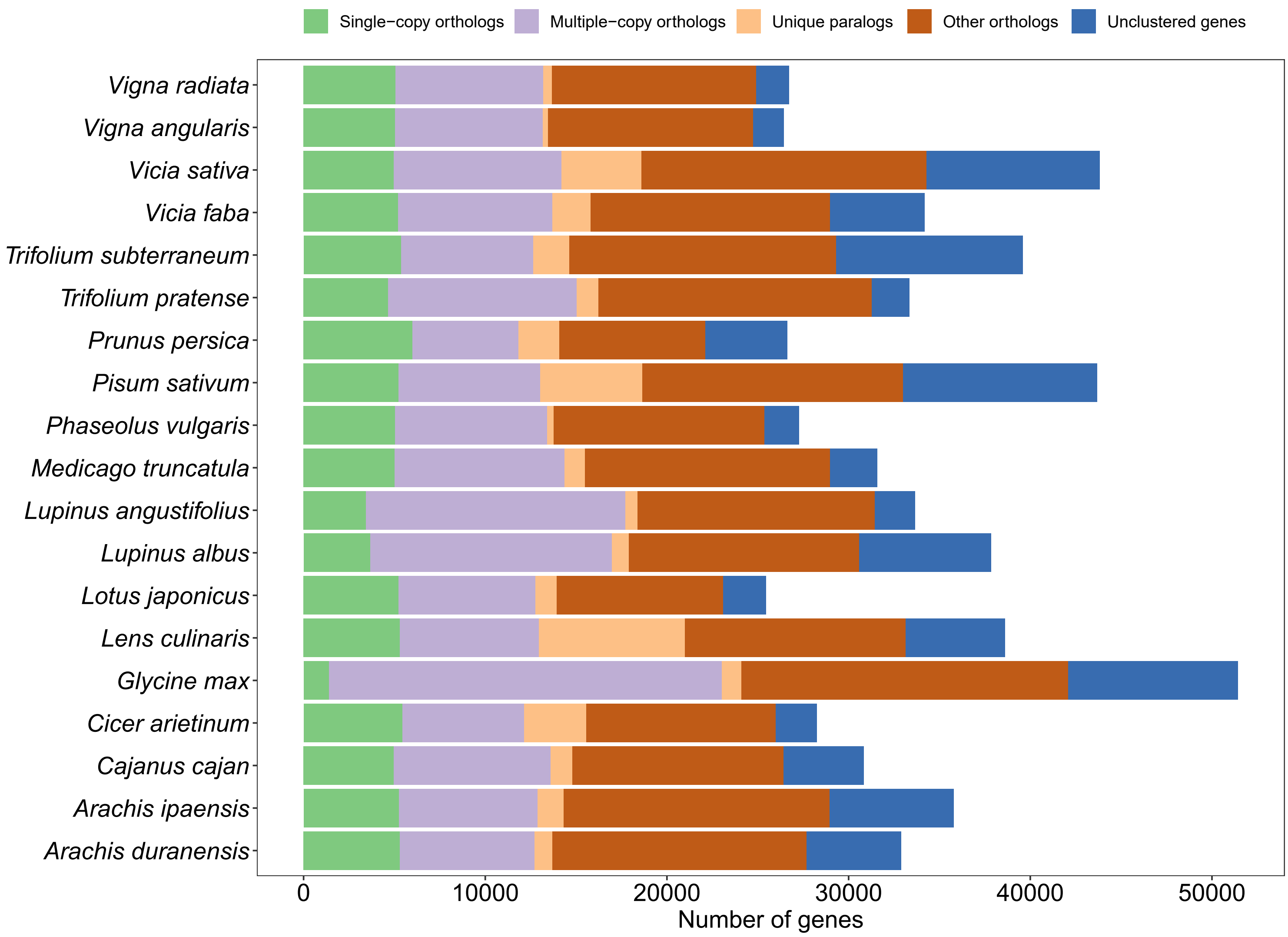

Supplementary Figure 2. A plot of homologous gene number in each of the 19 species. Single-copy orthologues, single-copy homologous genes in the gene families shared among species; multiple-copy orthologues, multicopy homologous genes in gene families shared among species; unique paralogues, genes of the strain unique to the family; other orthologues, all other genes; unclustered genes, genes not clustered into any family. The horizontal bars represent the number of protein-coding genes.

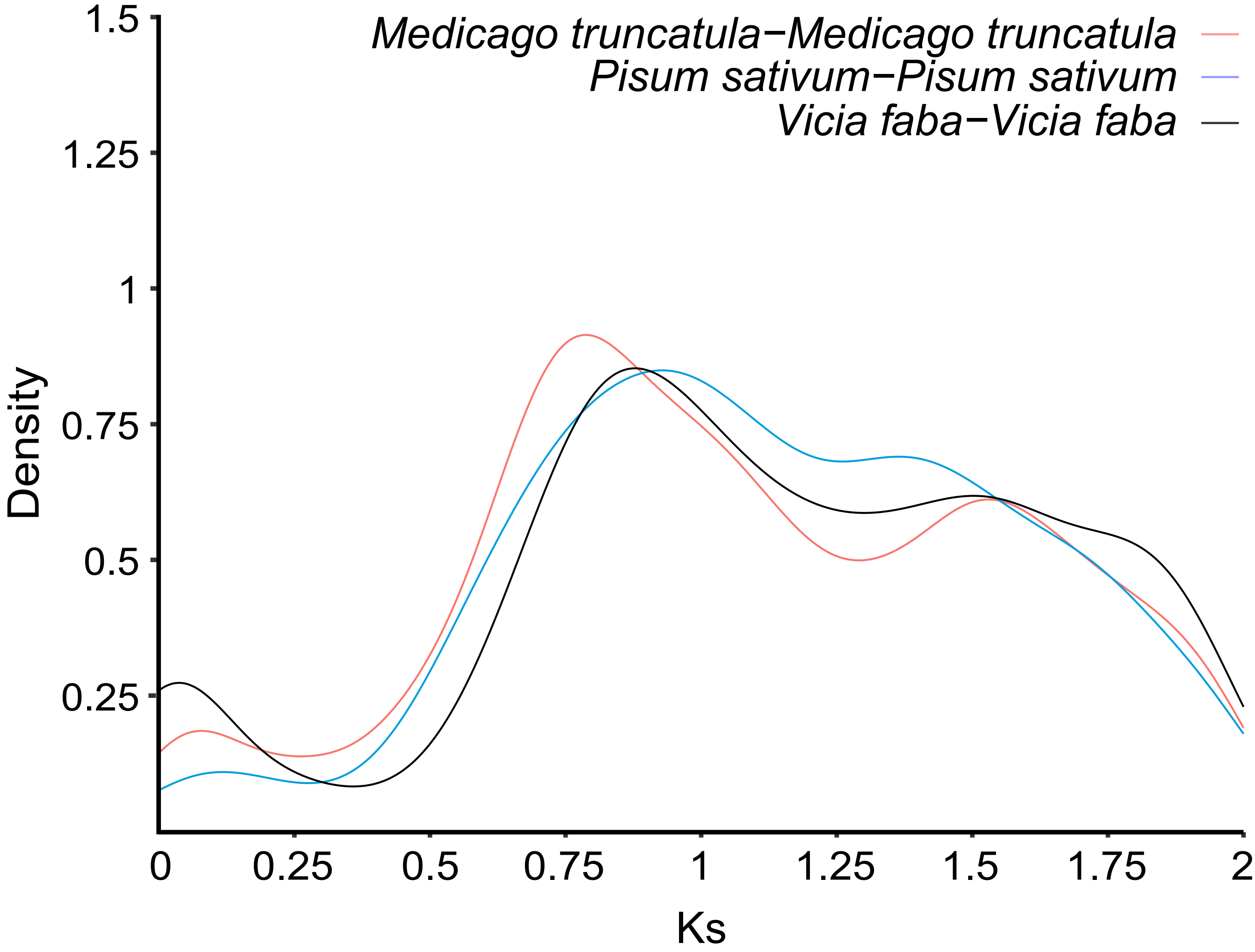

Supplementary Figure 3. Distribution of the synonymous substitution rate (Ks) between paralogous gene pairs of faba bean and other legumes.

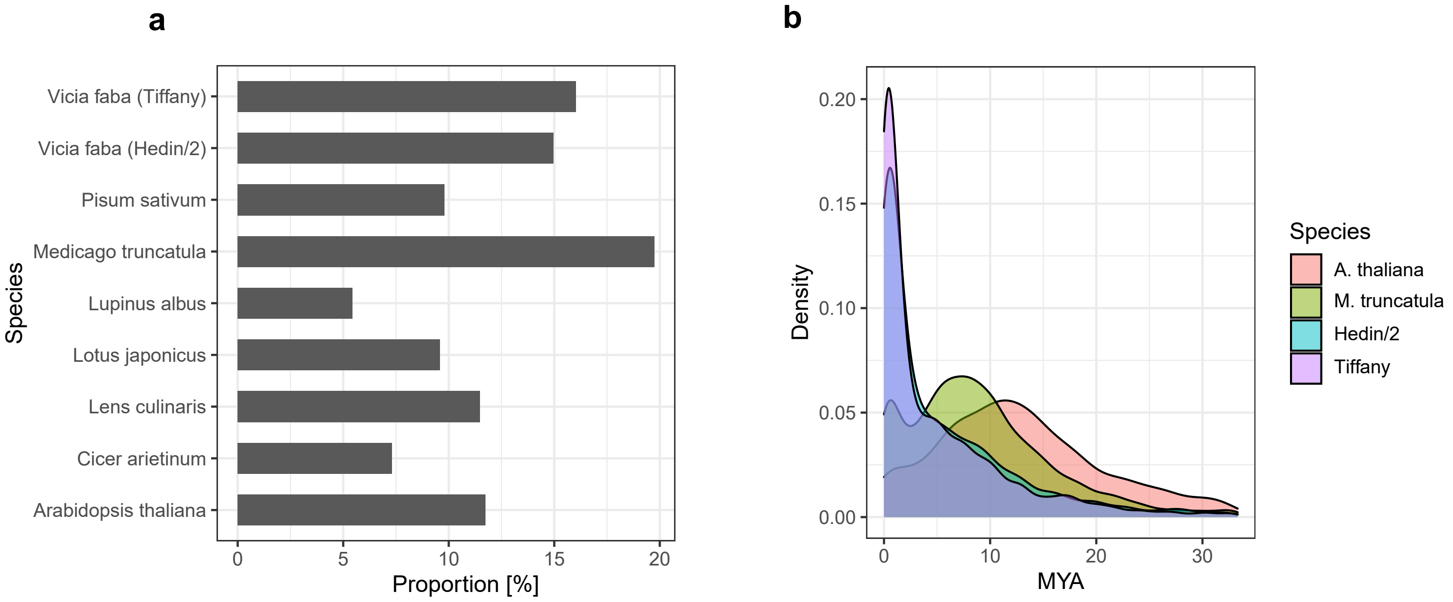

Supplementary Figure 4. **a**, Proportion of genes tandemly duplicated across species. **b**, Estimated age of tandem duplications.

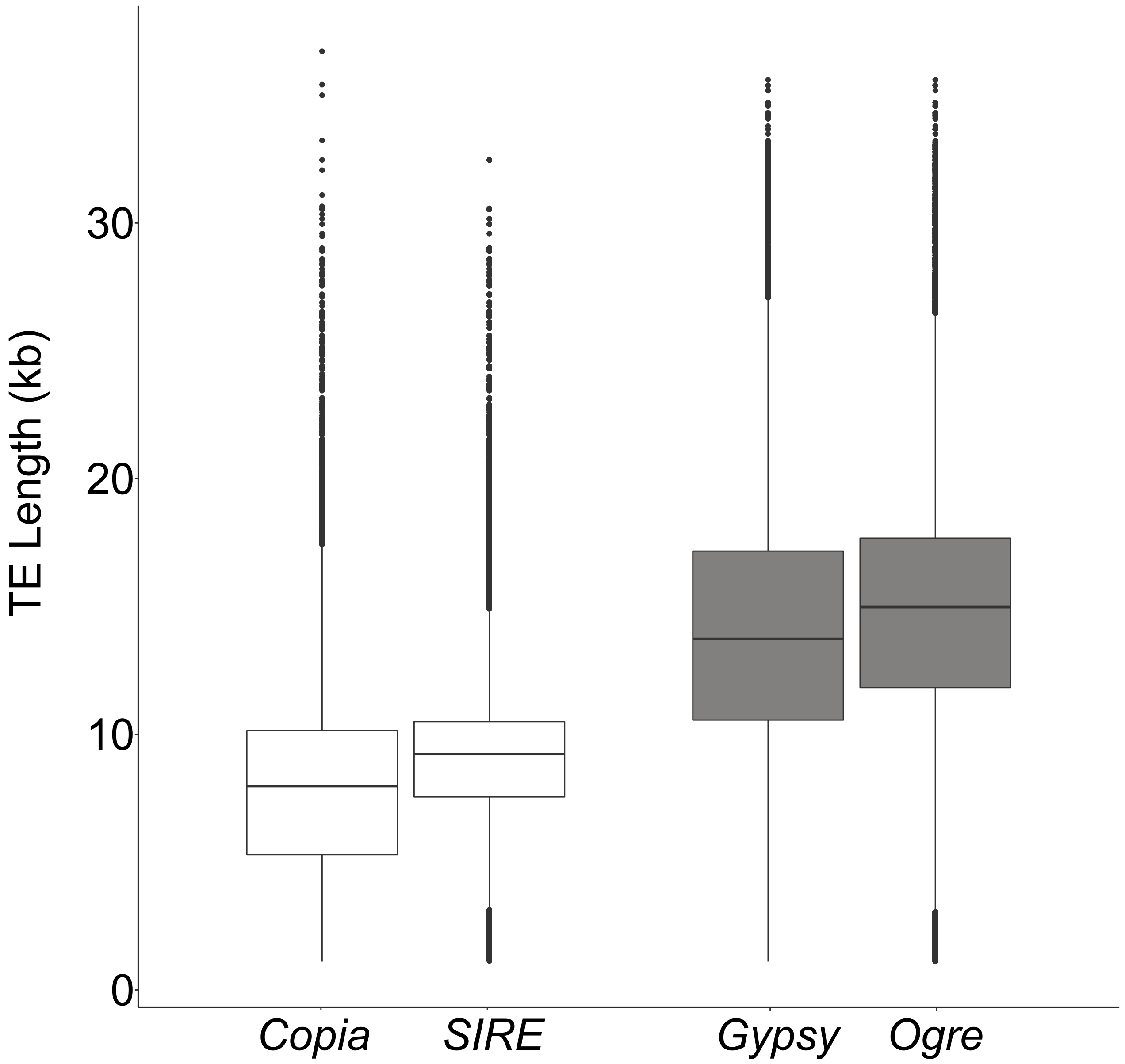

Supplementary Figure 5. Full-length representation of *Copia* and *Gypsy* super family. *Ogre* (n=2,157,340) and *SIRE* (n=918,214) are the largest elements in *Gypsy* (n=2,564,151) and *Copia* (n=1,578,228) respectively. centre line, median; box limits, upper and lower quartiles; whiskers, 1.5× interquartile range; points, outliers

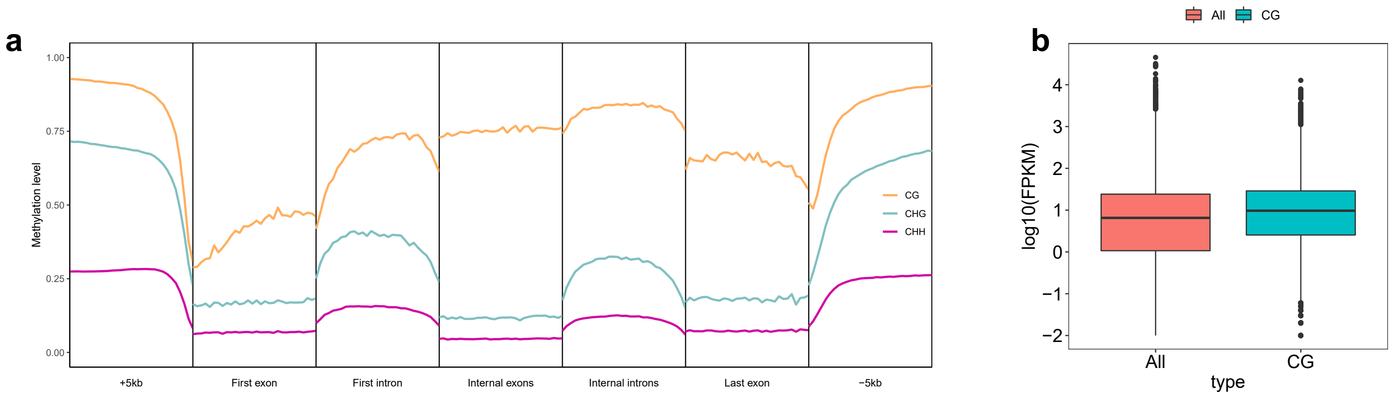

Supplementary Figure 6. **a,** Methylation pattern based on gene features including first exon, first intron, last exon, last intron, and the 5-kb flanking regions in the Hedin/2 genome. **b**, Expression comparison between all annotated genes and gene-body methylated genes (CG context) in young leaf tissue. All: n=34,221, CG: n=12,453. centre line, median; box limits, upper and lower quartiles; whiskers, 1.5× interquartile range; points, outliers

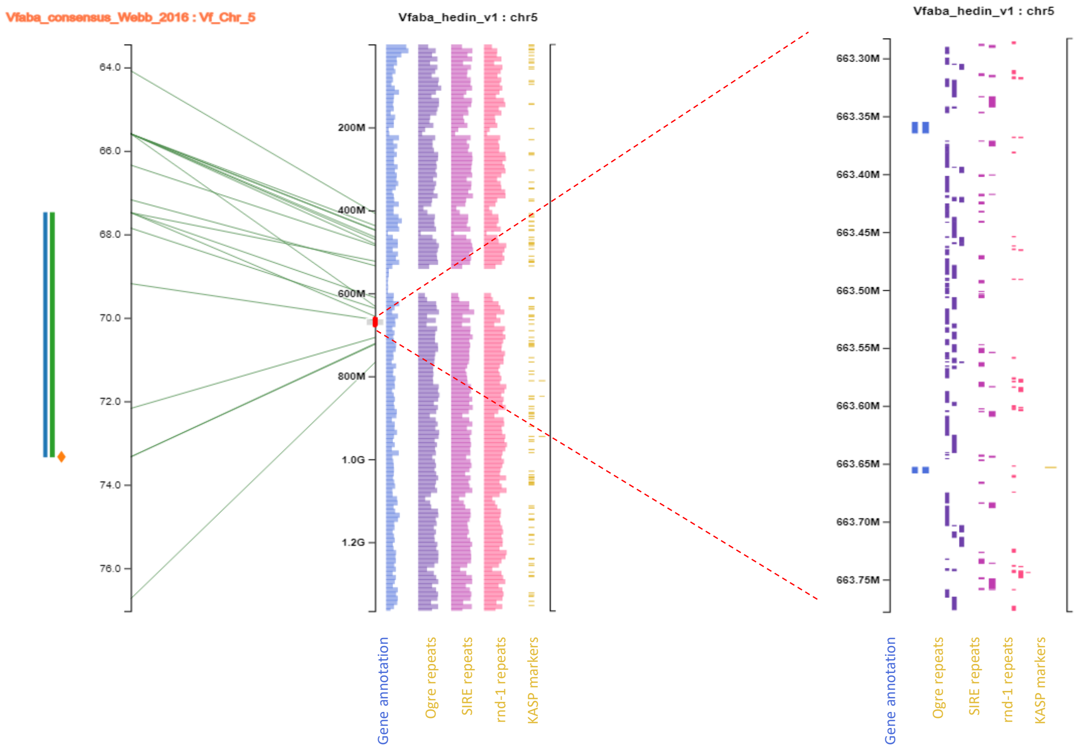

Supplementary Figure 7. Pretzel (pulses.plantinformatics.io) visualisations integrating the consensus genetic map from Webb et al. (2016) together with 3 QTLs for frost tolerance from Sallam et al. (2016) (left axis), aligned to the Hedin/2 genome (middle and right axes). The gene annotation for Hedin/2 is shown together with the three most abundant repeat types (*Ogre*, *SIRE* and rnd-1). Pretzel enables rapid, interactive interrogation of multiple data types and integration of legacy knowledge with the new assemblies.

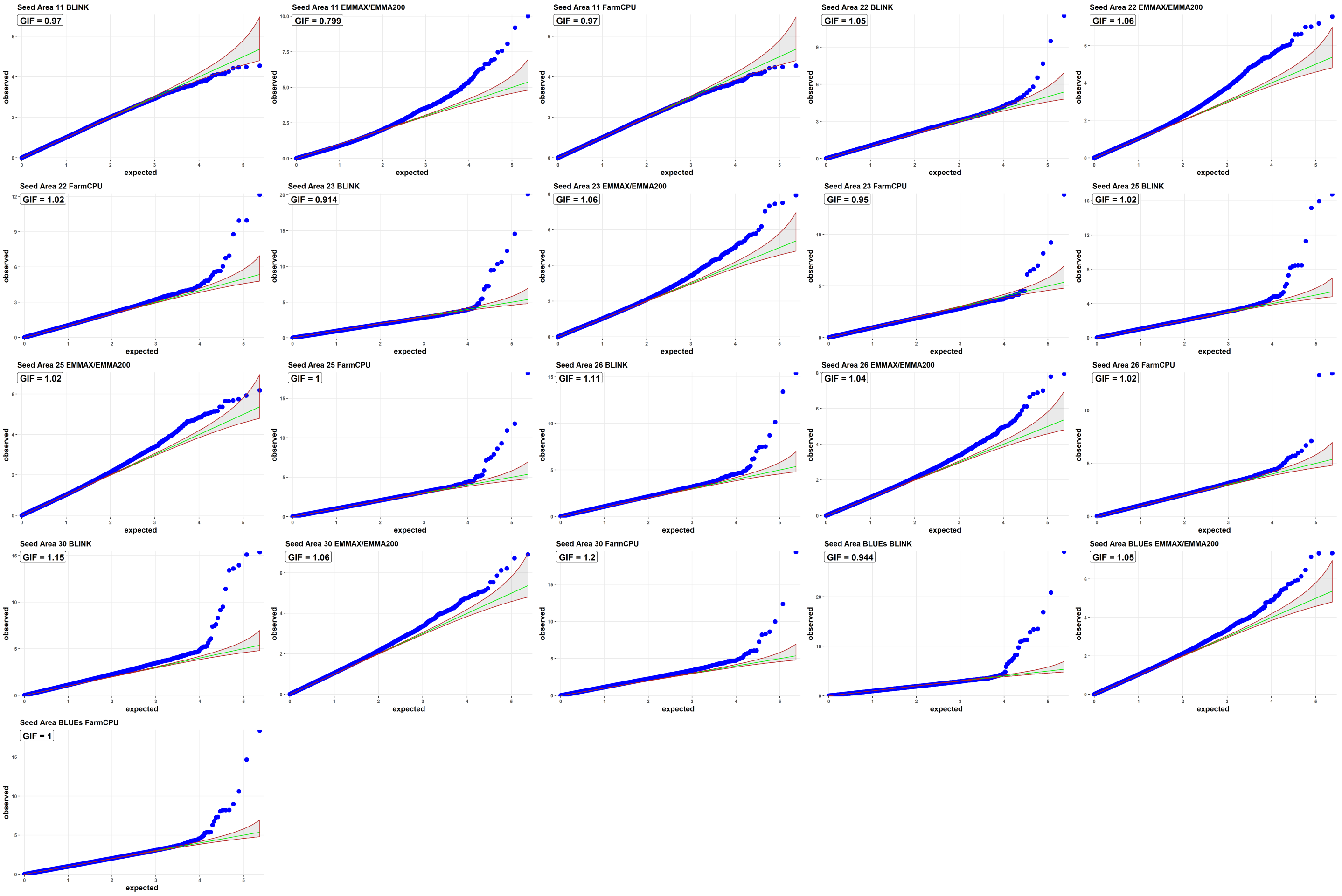

Supplementary Figure 8. Quantile-Quantile plots of seed area GWAS. Analysis was performed using three methods (BLINK, farmCPU, EMMAX) and phenotypes from six trials (11, 22, 23, 25, 26, 30). BLUE - best linear unbiased estimator across trials.

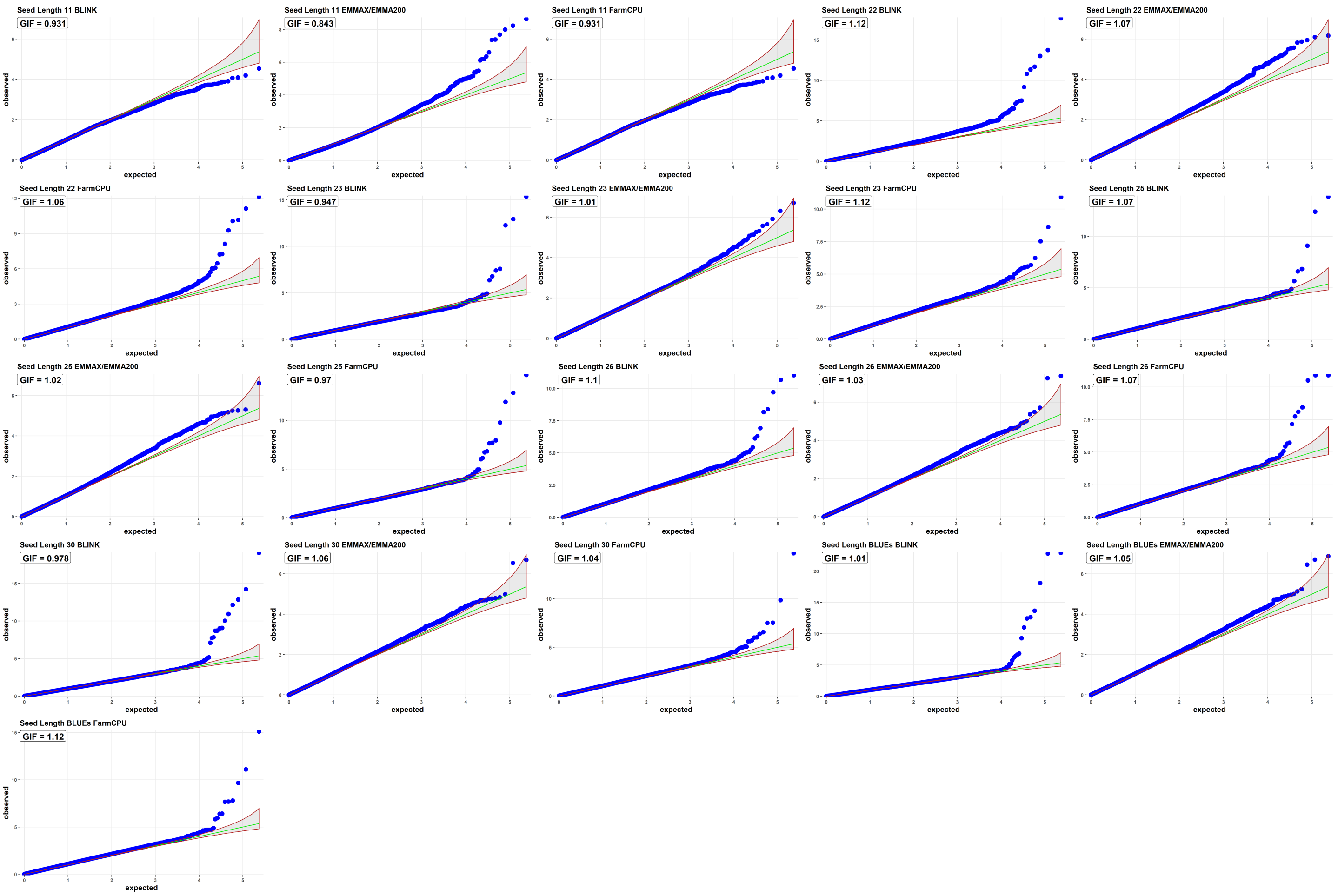

Supplementary Figure 9. Quantile-Quantile plots of seed length GWAS. Analysis was performed using three methods (BLINK, farmCPU, EMMAX) and phenotypes from six trials (11, 22, 23, 25, 26, 30). BLUE - best linear unbiased estimator across trials.

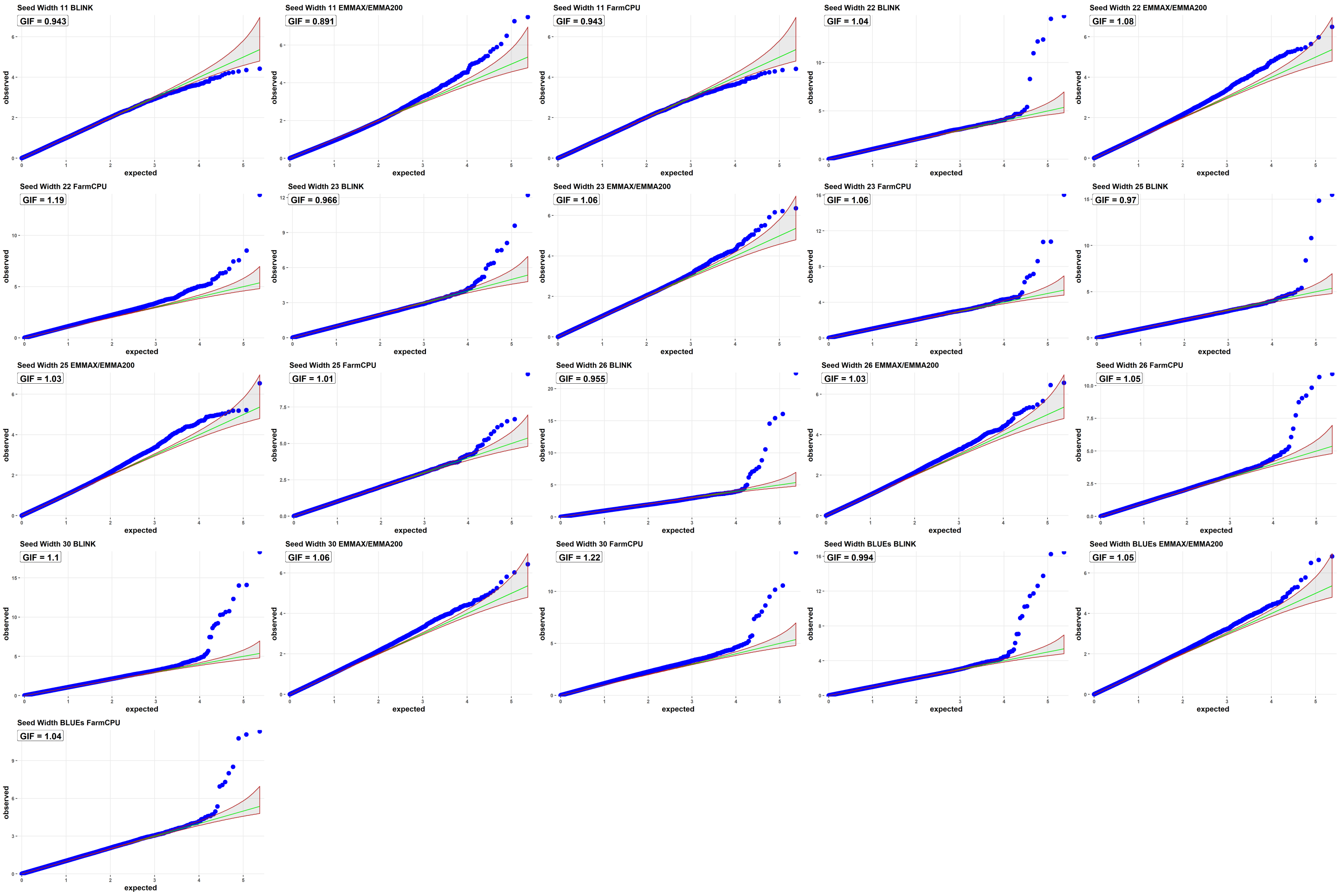

Supplementary Figure 10. Quantile-Quantile plots of seed width GWAS. Analysis was performed using three methods (BLINK, farmCPU, EMMAX) and phenotypes from six trials (11, 22, 23, 25, 26, 30). BLUE - best linear unbiased estimator across trials.

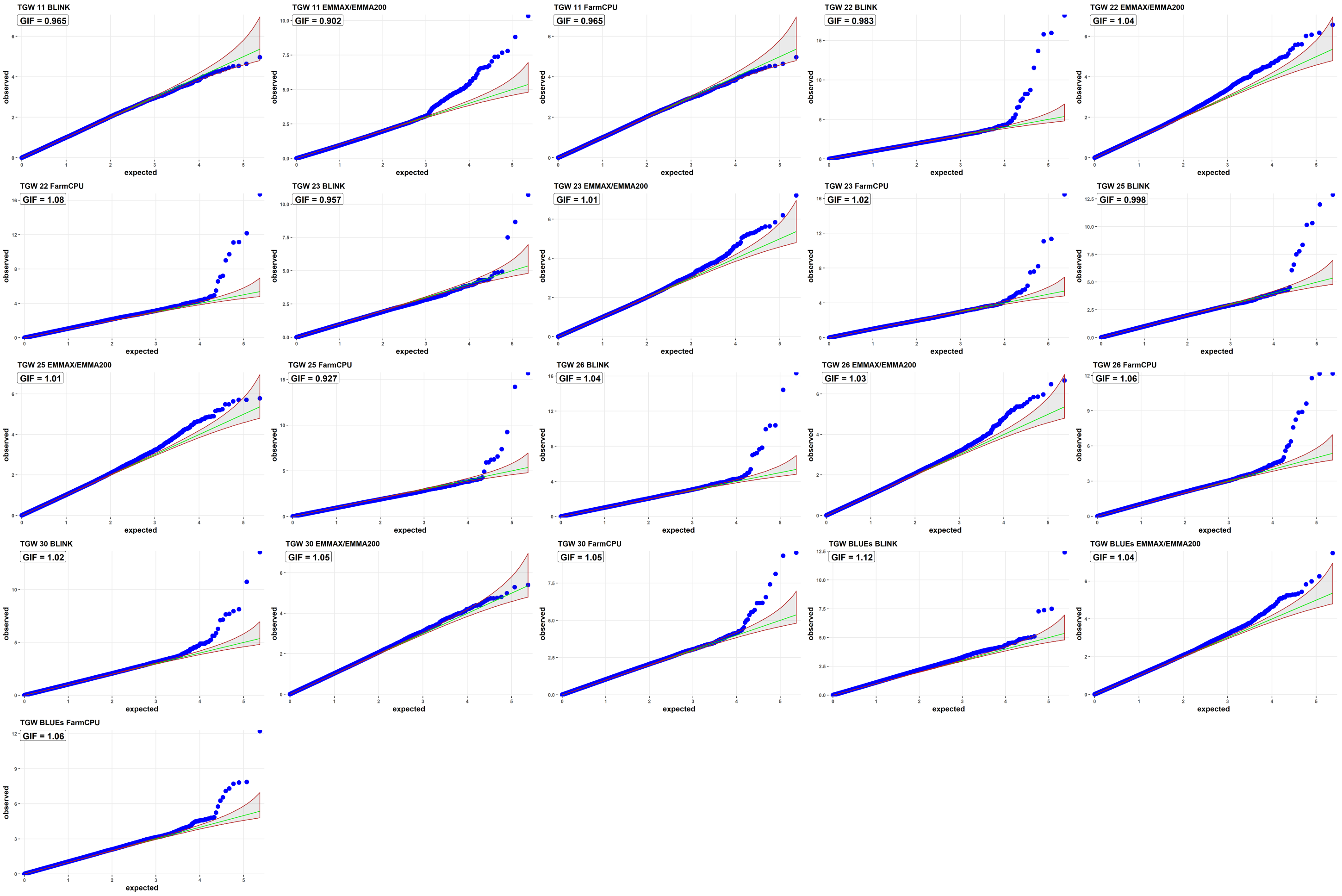

Supplementary Figure 11. Quantile-Quantile plots of thousand grain weight (TGW) GWAS. Analysis was performed using three methods (BLINK, farmCPU, EMMAX) and phenotypes from six trials (11, 22, 23, 25, 26, 30). BLUE - best linear unbiased estimator across trials.

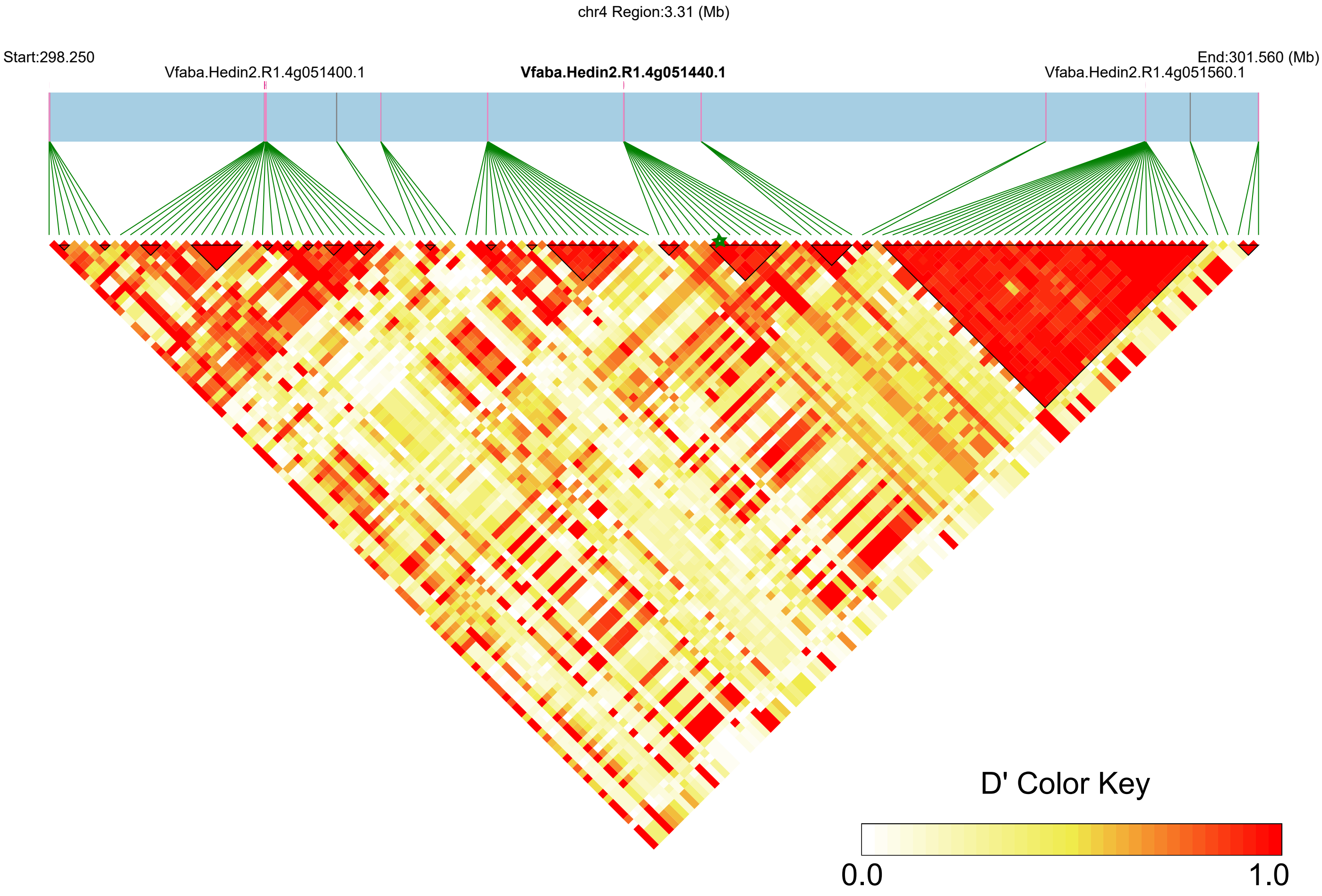

Supplementary Figure 12. LD patterns in the genomic region surrounding SNP chr4_299823118 highly associated with seed size and located in gene Vfaba.Hedin2.R1.4g051440 (indicated in bold) orthologous to AtCYP78A genes. Green lines indicate SNP positions. Green star indicates significant SNP.

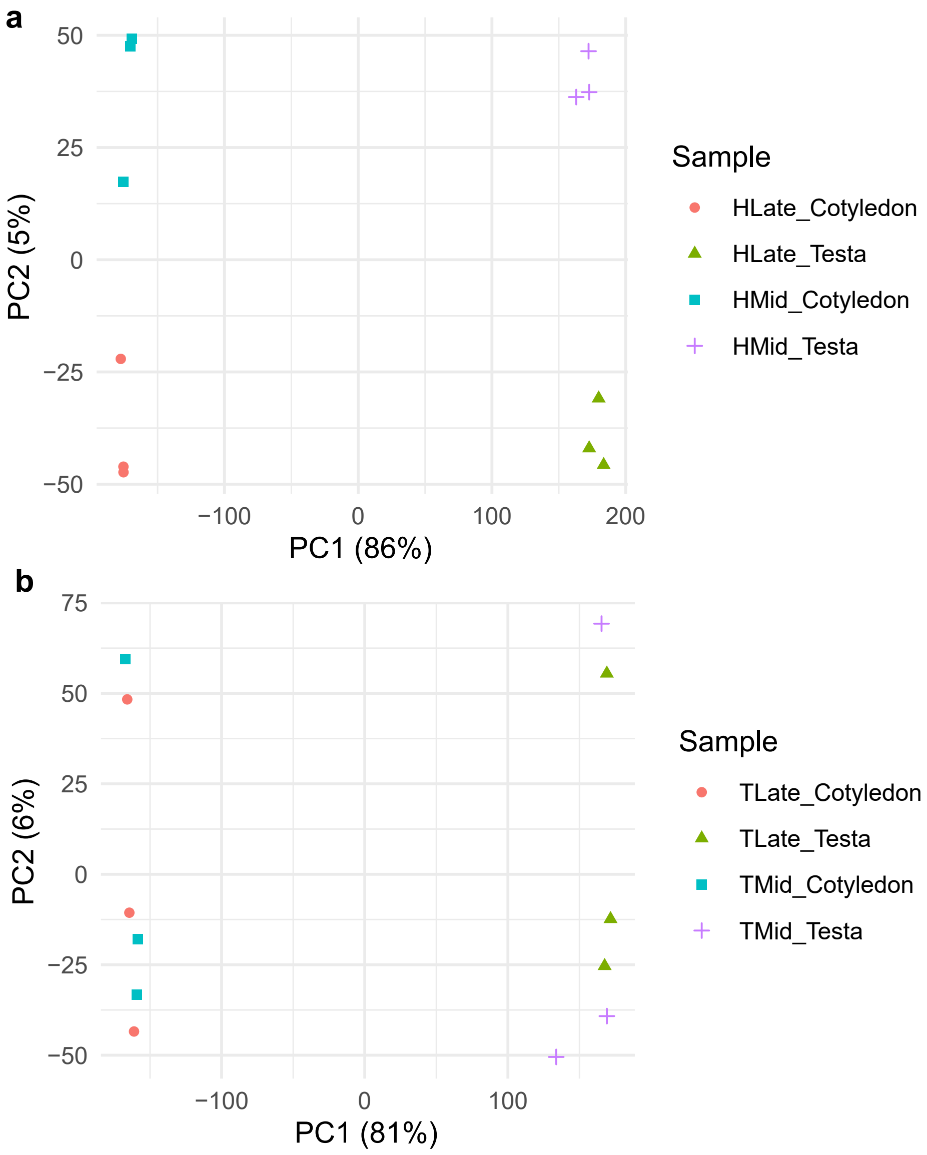

Supplementary Figure 13. PCA of libraries showing tissue and developmental stage differentiation. **a**, Hedin/2 libraries. **b**, Tiffany libraries.

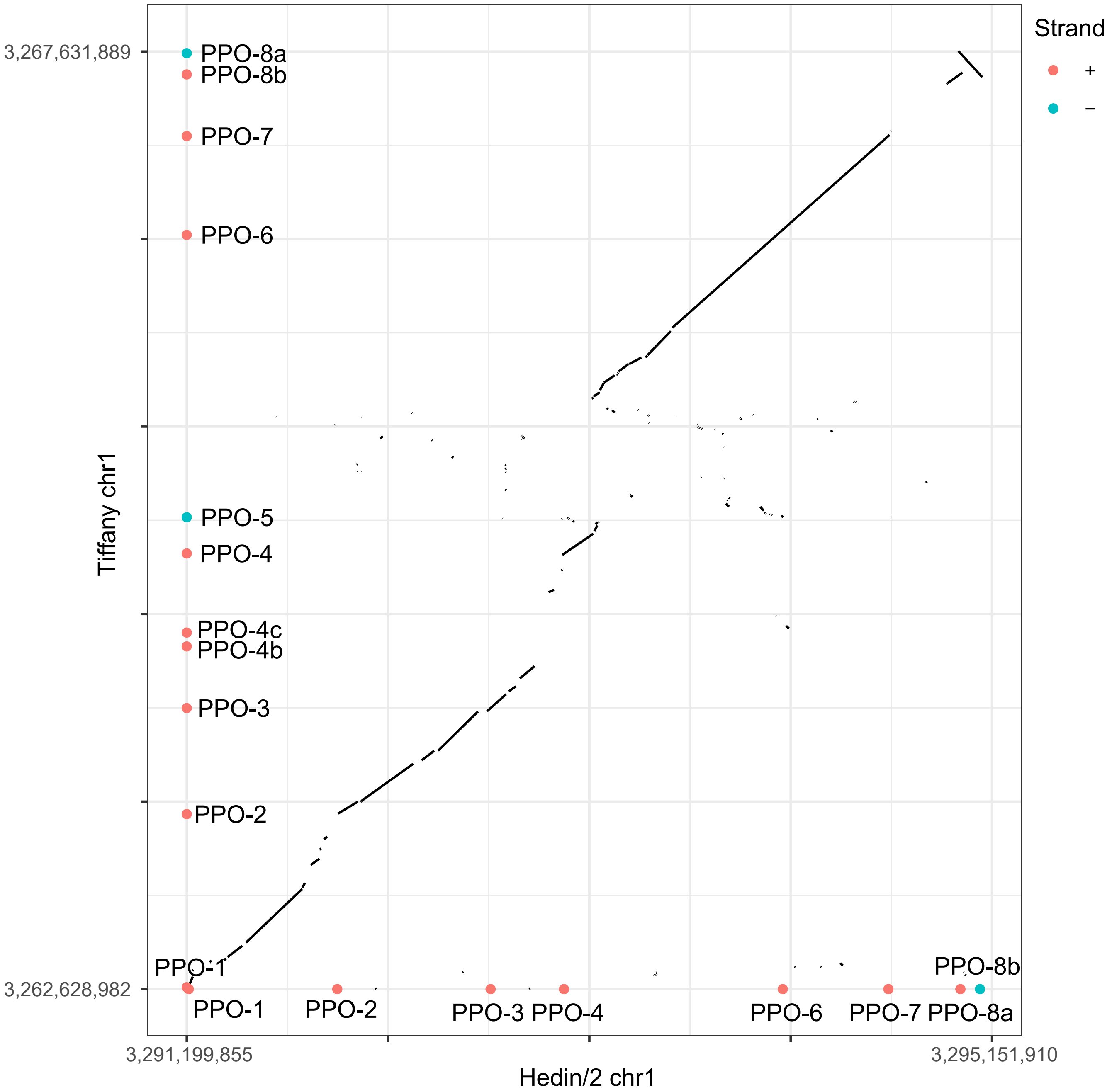

Supplementary Figure 14. A dot plot alignment of PPO locus of Hedin/2 and Tiffany. The red and blue dots represent genes coloured according to strand.

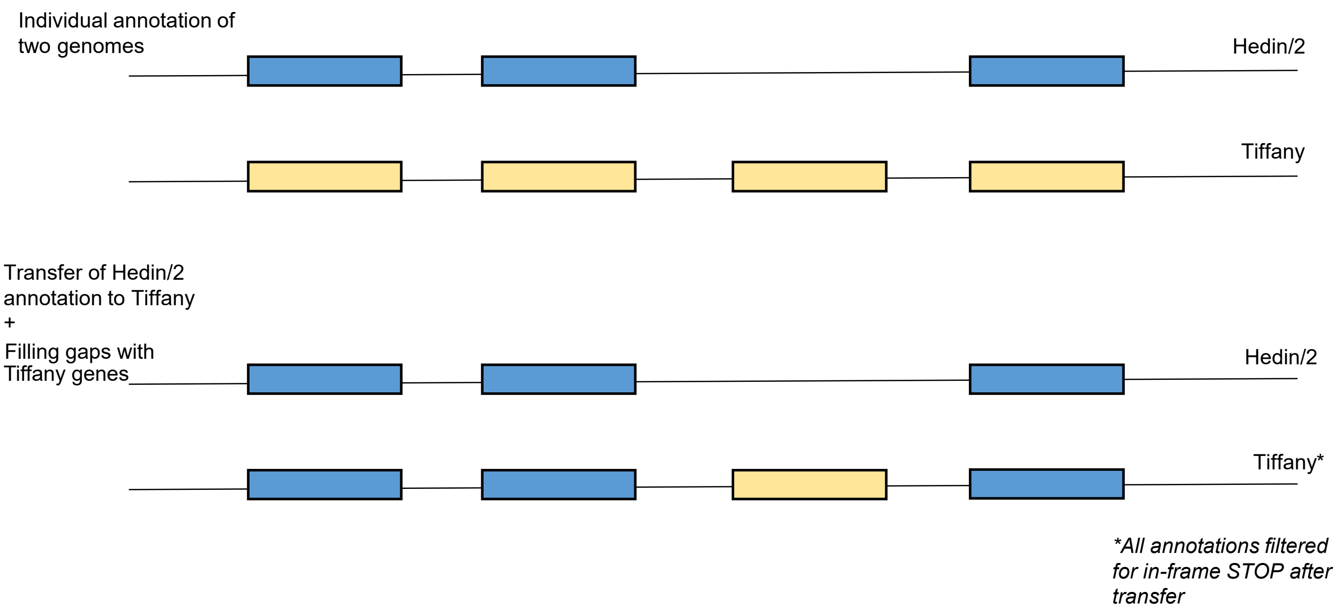

Supplementary Figure 15. Principle behind individual annotations and ‘transfer and gap fill’ strategy. Hedin/2 annotation was transferred onto Tiffany using Liftoff. To prevent formation of chimeric gene models, caused for example by SVs, transferred models with in-frame stop codons were removed and replaced by Tiffany genes. Gene models unique to Tiffany were also added to the annotation. Overall, we observed that the 'transfer and gap fill' approach resulted in more syntenic genes and more genes with the same CDS length in both accessions.

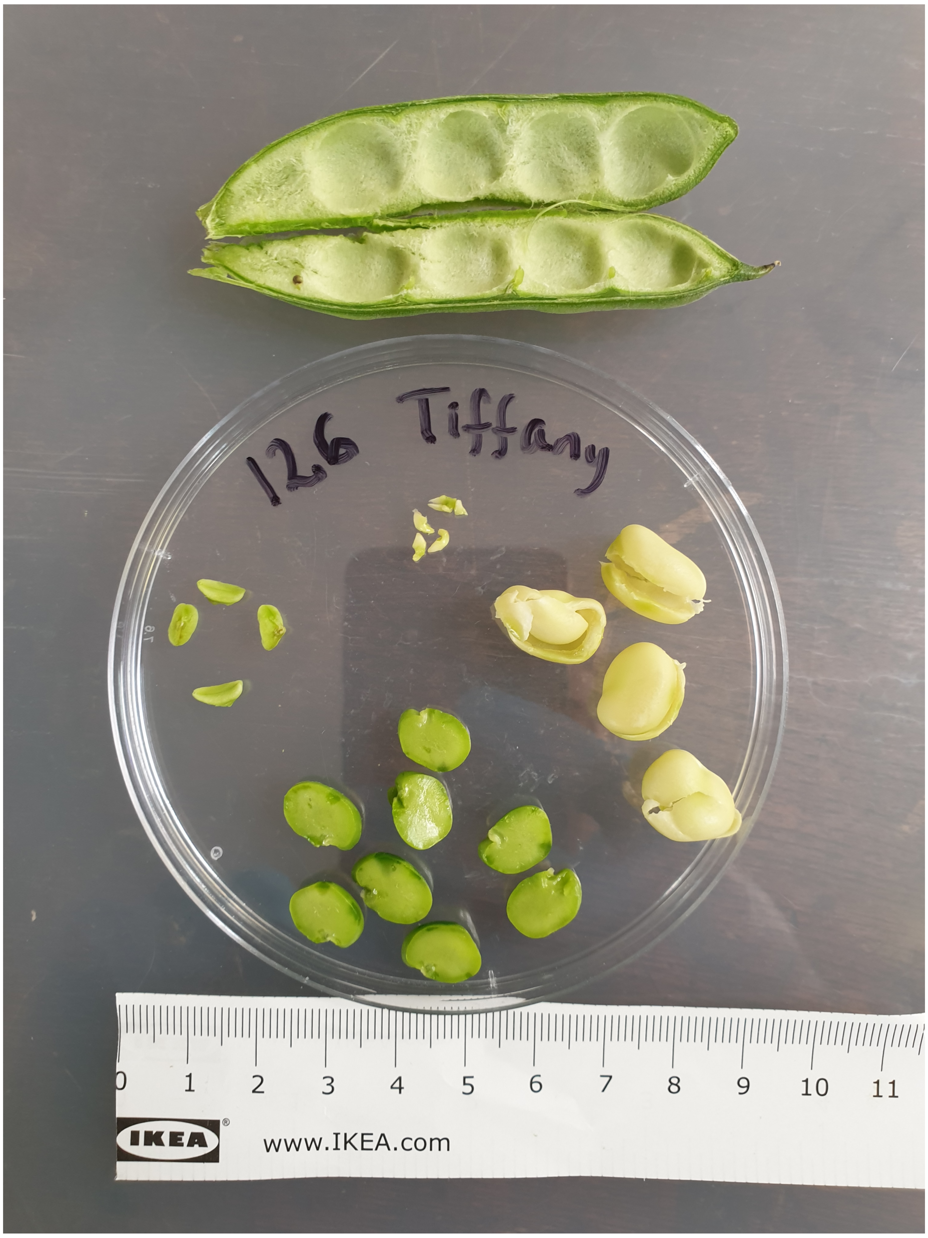

Supplementary Figure 16. Dissected parts of a pod of cv Tiffany: pod wall, testa, cotyledon, funiculus and embryo axis.

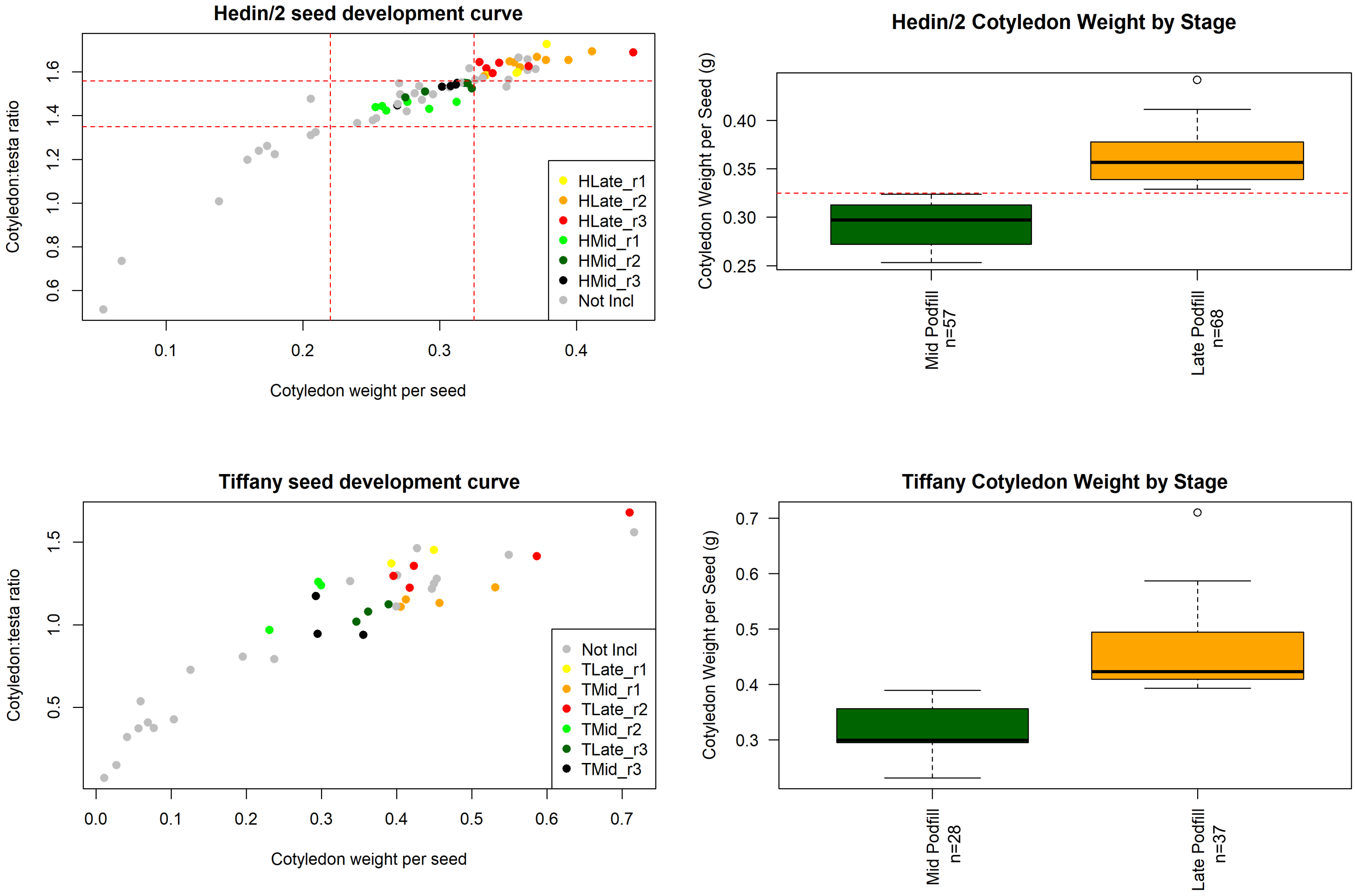

Supplementary Figure 17. Composition and phenotypic differentiation of testa and cotyledon libraries from a developmental pod-fill gradient. centre line, median; box limits, upper and lower quartiles; whiskers, 1.5× interquartile range; points, outliers

Supplementary Table 3. Structural annotation statistics for Hedin/2 and Tiffany assemblies

Annotation	Gene total length	Gene average length	Gene number	Exon total length	Exon average length	CDS average length
Hedin/2 all (coding + lncRNA)	104,751,785	2,357	44,439	42,500,136	224	1,163
Hedin/2 complete (coding with complete CDS only)	95,042,832	2,777	34,221	39,546,400	231	1,164
Tiffany complete (coding with complete CDS only)	95,719,908	2,812	34,043	38,674,818	226	1,148

Supplementary Table 4. Annotation BUSCO completeness. Complete: gene length within two standard deviations of the BUSCO group mean length. Duplicated: Complete genes found with more than one copy. Fragmented: genes only partially recovered. Missing: genes not recovered. Groups checked: resolution

**Annotation**	**Complete** [S:Single copy, D:Duplicated]	**Fragmented**	**Missing**	**Groups checked**	**BUSCO database**
Hedin/2 all	96.8%[S:78.0%,D:18.8%]	2.0%	1.2%	255	embryophyta_odb10
	94.7%[S:90.6%,D:4.1%]	0.9%	4.4%	5366	fabales_odb10
Hedin/2 complete	96.8%[S:78.0%,D:18.8%]	2.0%	1.2%	255	embryophyta_odb10
	94.5%[S:90.4%,D:4.1%]	0.9%	4.6%	5366	fabales_odb10
Tiffany complete	94.9%[S:77.3%,D:17.6%]	2.7%	2.4%	255	embryophyta_odb10
	90.2%[S:85.7%,D:4.5%]	1.0%	8.8%	5366	fabales_odb10

Supplementary Table 6. Summary statistics of gene families

**Species**	Gene number	Genes in families	Un-clustered genes	Family number	Unique families	Average genes per family
Arachis duranensis	32,899	27,703	5,196	17,302	341	1.6
Arachis ipaensis	35,785	28,950	6,835	17,438	573	1.66
Cajanus cajan	30,842	26,425	4,417	16,507	256	1.6
Cicer arietinum	28,260	25,996	2,264	15,181	322	1.71
Glycine max	51,431	42,082	9,349	17,633	396	2.39
Lens culinaris	38,624	33,149	5,475	16,672	929	1.99
Lotus japonicus	25,460	23,104	2,356	14,613	324	1.58
Lupinus albus	37,837	30,588	7,249	17,318	289	1.77
Lupinus angustifolius	33,663	31,447	2,216	16,644	200	1.89
Medicago truncatula	31,571	28,978	2,593	16,857	368	1.72
Phaseolus vulgaris	27,263	25,366	1,897	16,890	112	1.5
Pisum sativum	43,677	32,990	10,687	18,684	1,476	1.77
Prunus persica*	26,631	22,127	4,504	14,490	604	1.53
Trifolium pratense	33,342	31,284	2,058	17,108	388	1.83
Trifolium subterraneum	39,578	29,312	10,266	17,066	740	1.72
Vicia faba	34,221	28,970	5,211	17,721	700	1.63
Vicia sativa	43,831	34,301	9,530	18,275	1,246	1.88
Vigna angularis	26,445	24,753	1,692	16,506	111	1.5
Vigna radiata	26,715	24,917	1,798	16,511	144	1.51

*outgroup.

Supplementary Table 13. Markers which show stable associations with seed traits

Trait	Reference	Alternative	Seed enlarging allele	Chromosome	Position
Seed Width/Length/Area	T	C	ALT	1S	154,878,079
Seed Width/Area	G	C	ALT	1S	658,098,019
Seed Length/TGW	A	T	ALT	1L	1,535,386,197
Seed Width/Area	A	C	ALT	1L	562,543,737
Seed Area/Width	G	A	ALT	2	1,335,507,680
Seed Width/TGW	A	T	REF	3	88,655,178
Seed Length/Area	C	G	ALT	3	165,271,925
Seed Area/Width	C	T	ALT	4	299,823,118
Seed Length/Area	G	C	ALT	4	620,271,647
Seed Width/Length	G	C	ALT	4	586,613,628
Seed Area/TGW	T	C	ALT	5	497,436,071
Seed Area/Width	T	C	ALT	5	615,661,639
TGW	G	A	ALT	5	968,403,536
Seed Length	G	T	ALT	6	1,019,941,571
Seed Length/Area	T	C	ALT	6	1,232,515,946

Supplementary Note: Pretzel instructions

The below steps demonstrate how to compare the seed weight QTL on chromosome 4 reported in Khazaei et al. 2014 with the GWAS result for Seed Area/Width reported in the present paper.

1. Log in

Access Pretzel Pulses URL: https://pulses.plantinformatics.io/

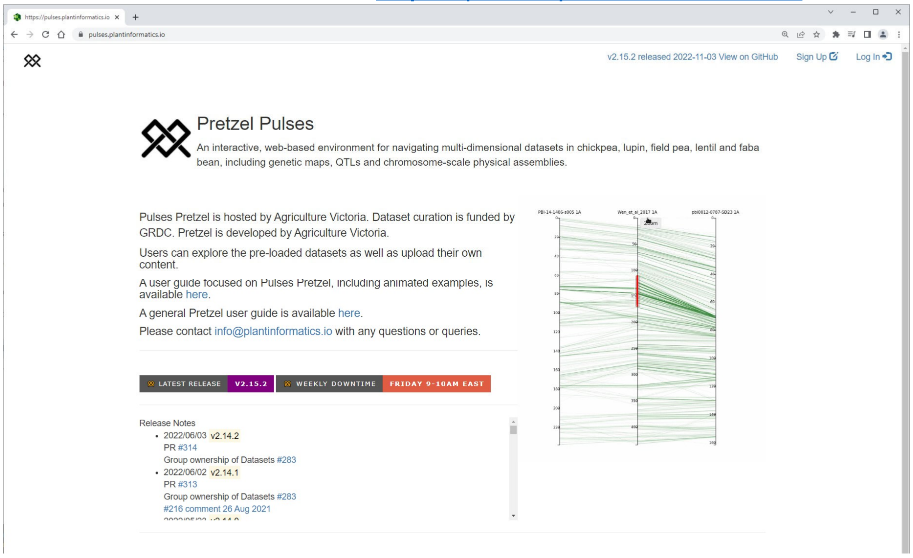

Click Log In in top right

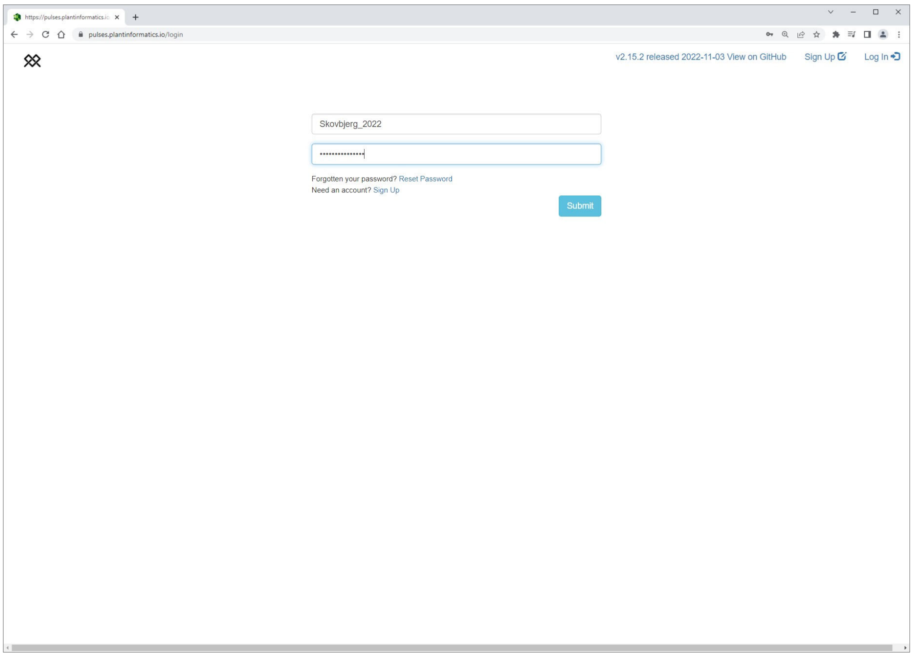

Log in with the following details:
Username: FabaBeanGenome
Password: ViciaFaba99

2. Search for markers of interest

Click the Search tab at the top of the left panel, and cut/paste the markers from Khazaei et al. 2014, Figure 3, around the seed weight QTLs reported on chromosome 4 (Vf_Mt8g020800_001, CNGC4, Vf_Mt7g038120_001, Vf_Mt8g040150_001) and click Search.

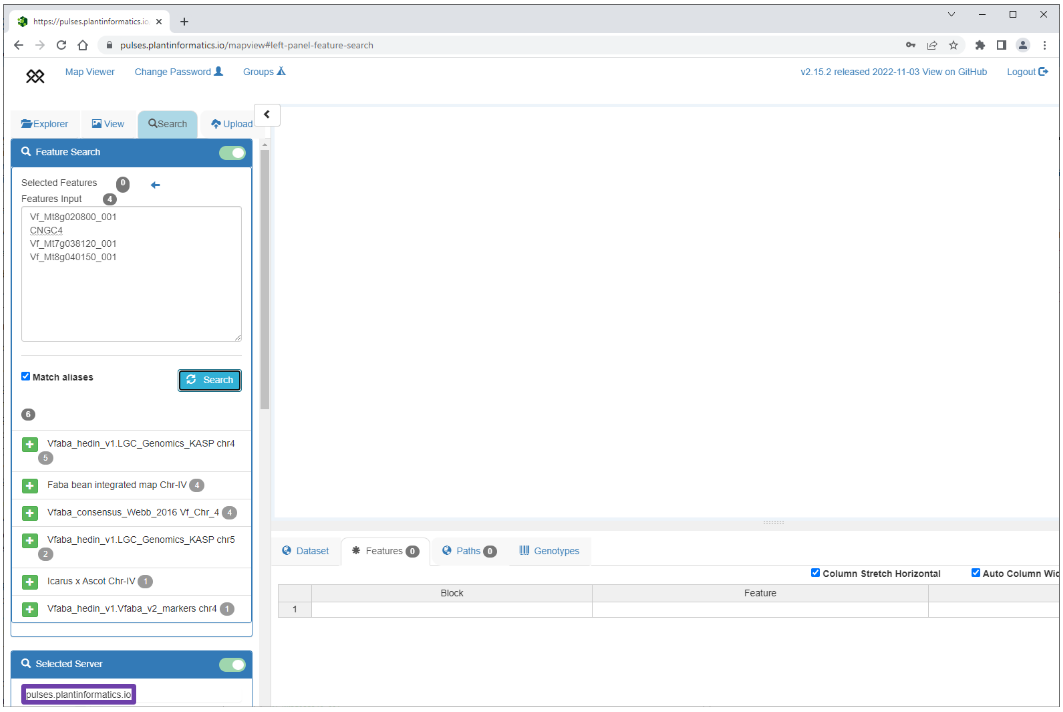

3. Add dataset to the view

Click the + button next to Faba bean integrated map Chr-IV

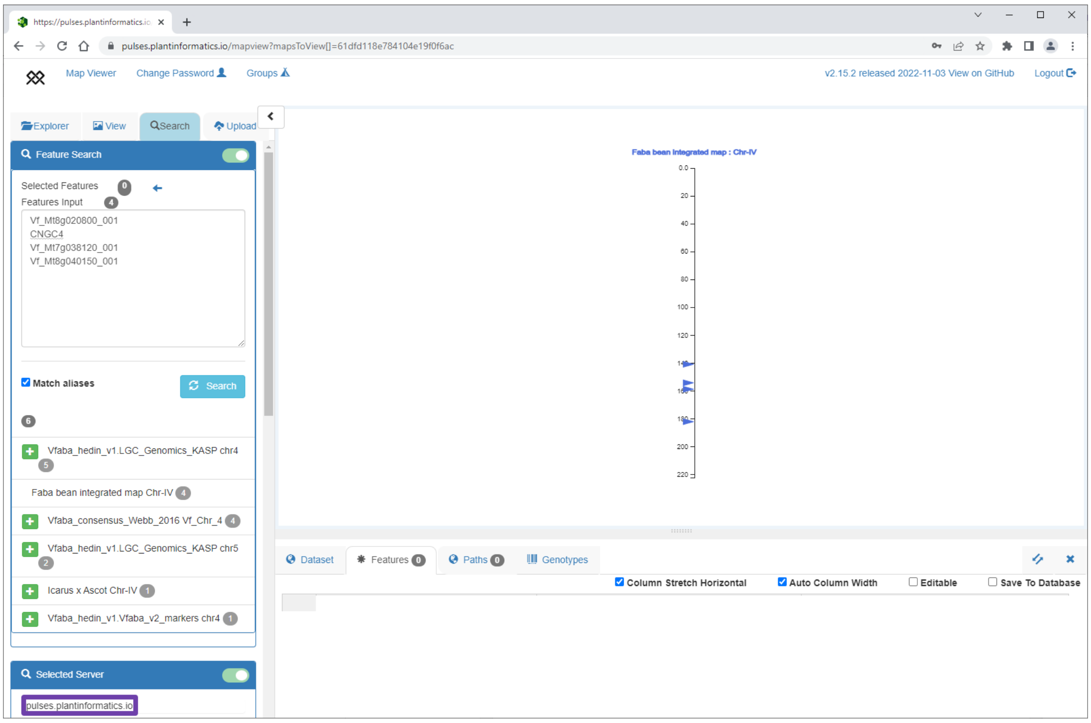

4. Add physical genome position with marker positions

Click on the Explorer tab on the left side panel.

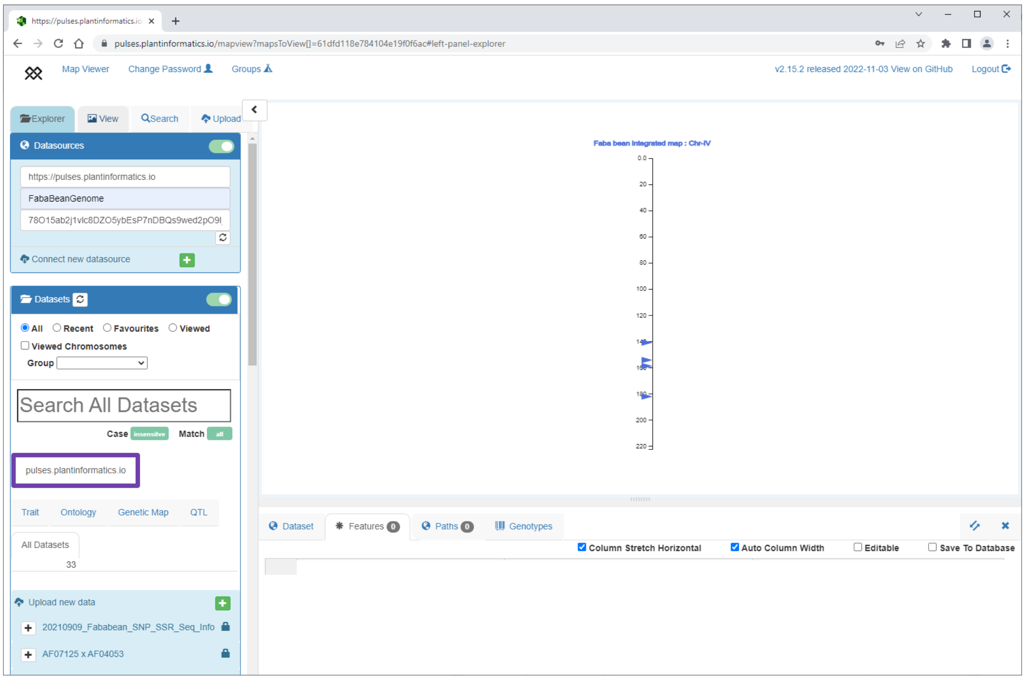

Add the following dataset:

- Vfaba_hedin_v1.Vfaba_v2_markers – chr4

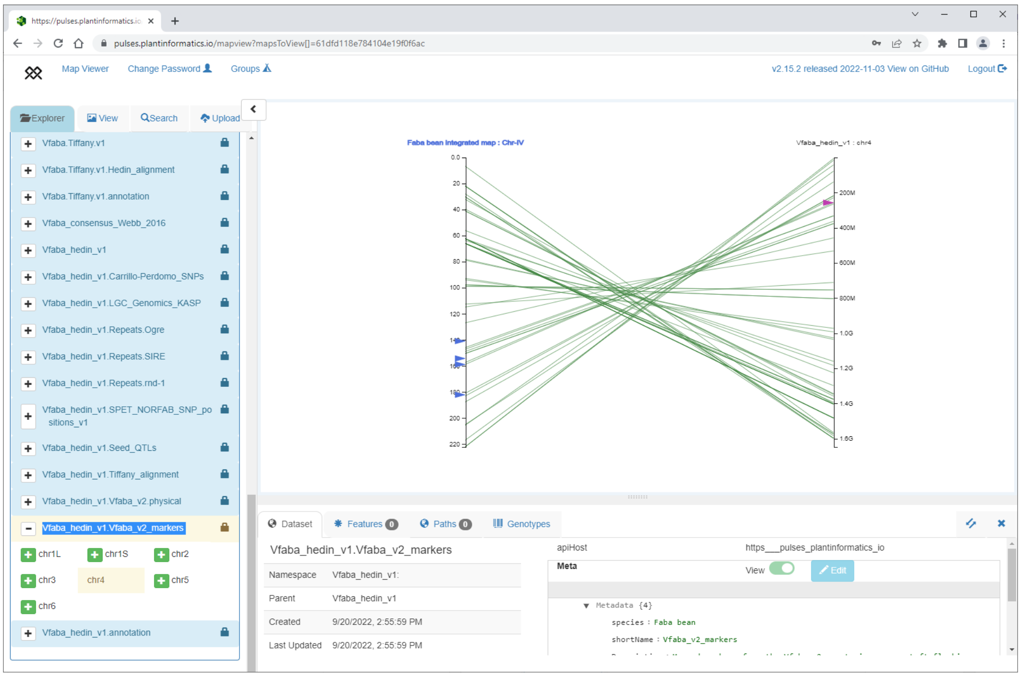

The genetic map is now projected into the physical chromosome assembly.

5. Reverse the orientation of the inverted genetic consensus map

Click the text (Faba bean integrated map : Chr-IV) at the top of the left axis to open the axis menu. Click the button with up/down arrows to invert the axis.

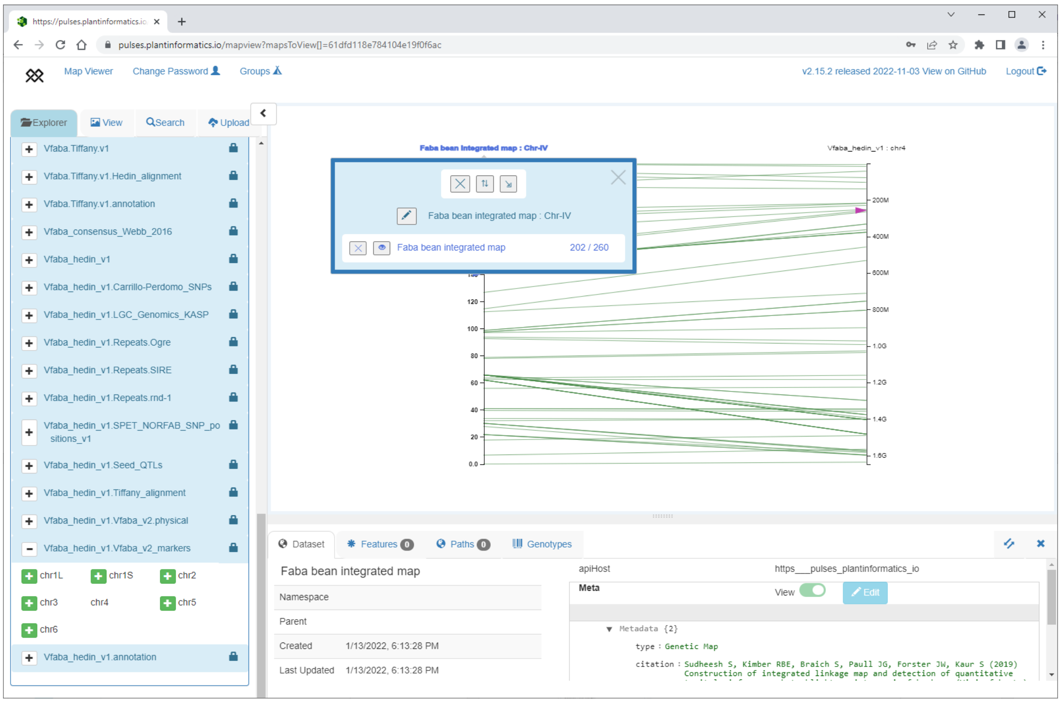

Click the “x” in the top right of the axis menu to close it.

6. Add Jayakodi et al. 2022 GWAS results to the view

Select the following dataset from the list in the left panel:

- Vfaba_hedin_v1.Jayakodi2022 - chr4

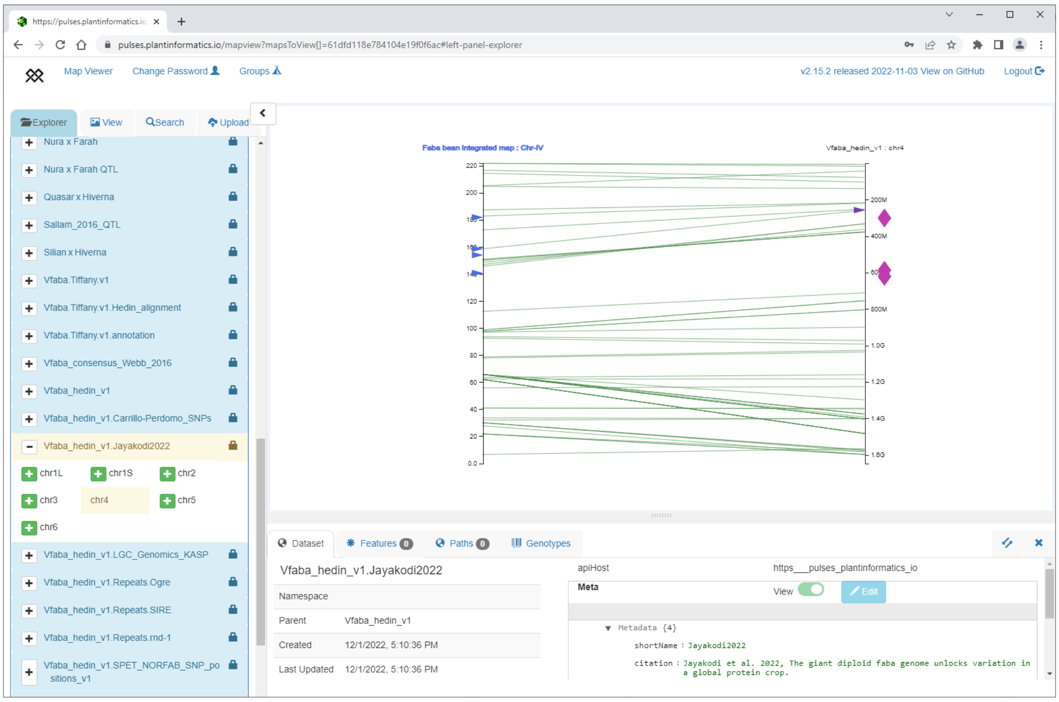

7. Colour GWAS peak markers by trait

In the View tab of the left panel, select Trait.

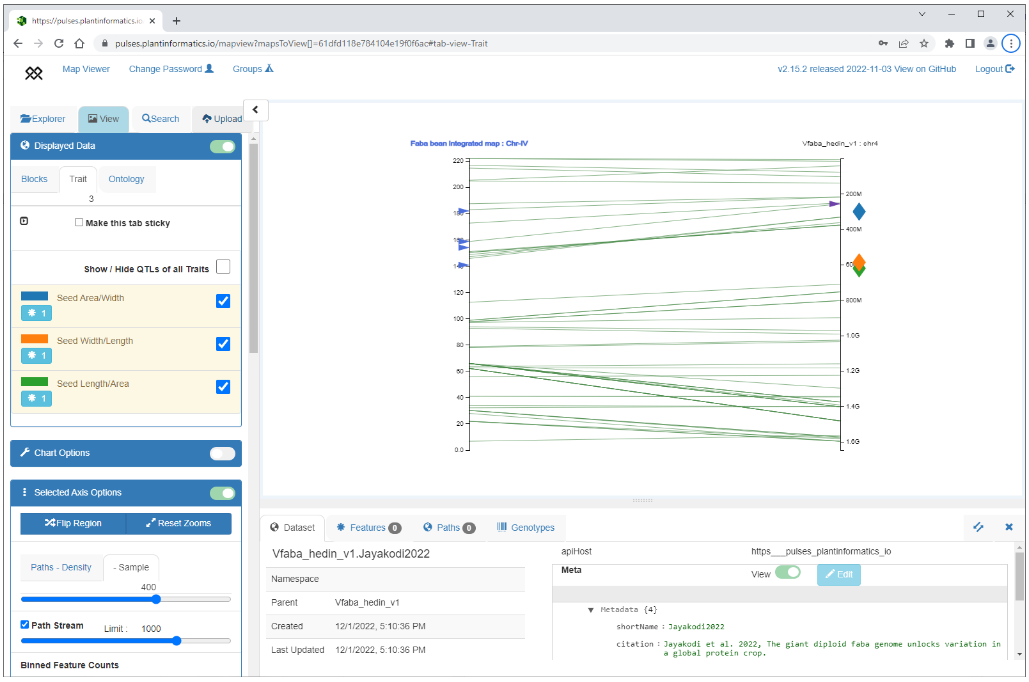

8. Annotate the view with marker names

Click the triangles on the left axis to add the marker name to the visualisation.

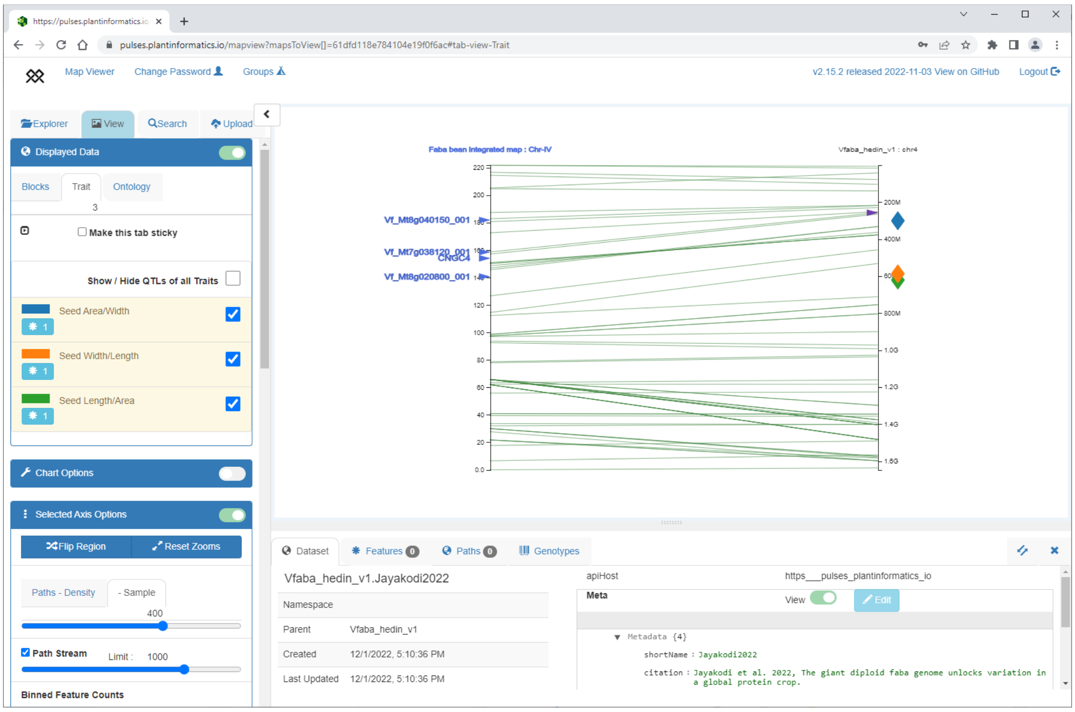

9. Zoom in to region of interest

Click and drag on the region of interest to create a brush.

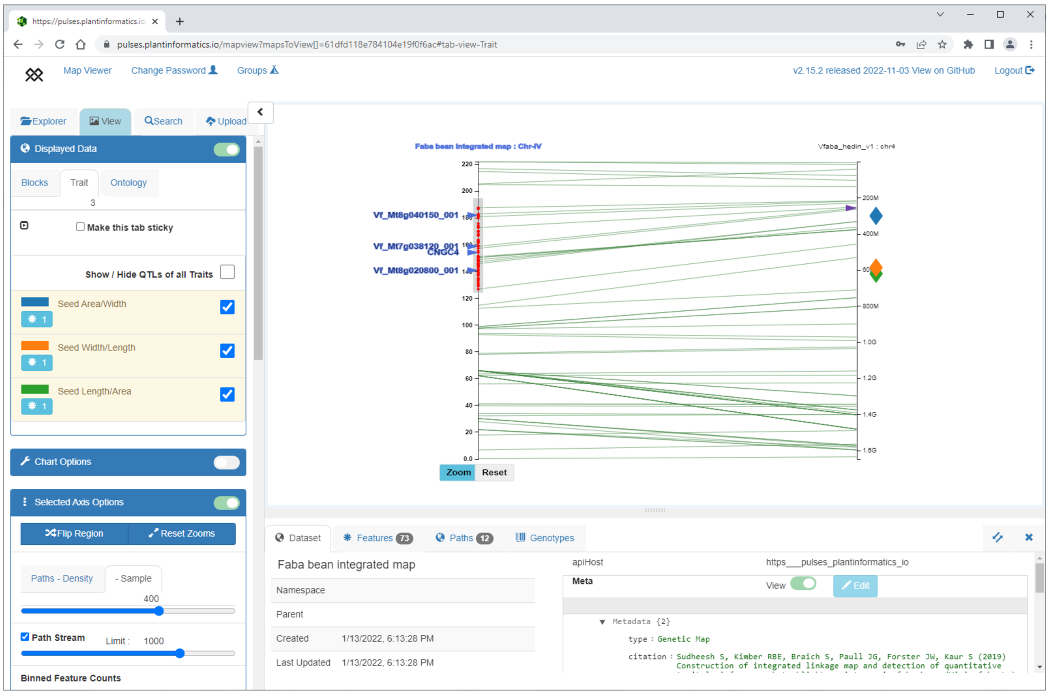

Click the Zoom button to zoom the axis to the region defined by the brush.

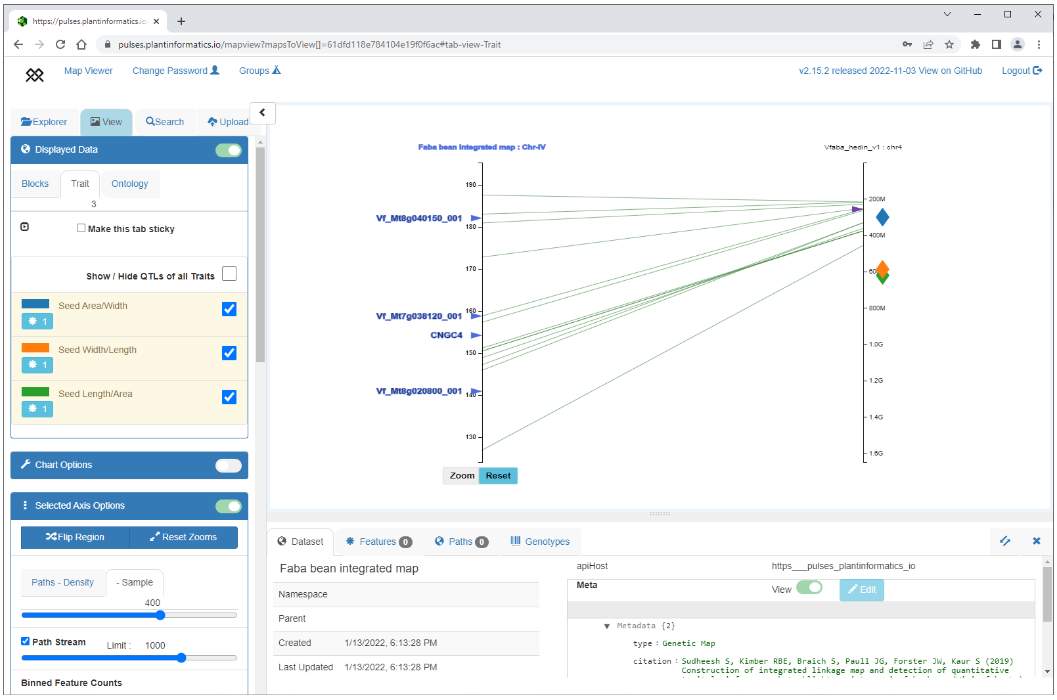

Zoom in to the right axis in the same way. Click the diamond on the side of the right axis to add a triangle at that position, then click the triangle to add the name of the QTL.

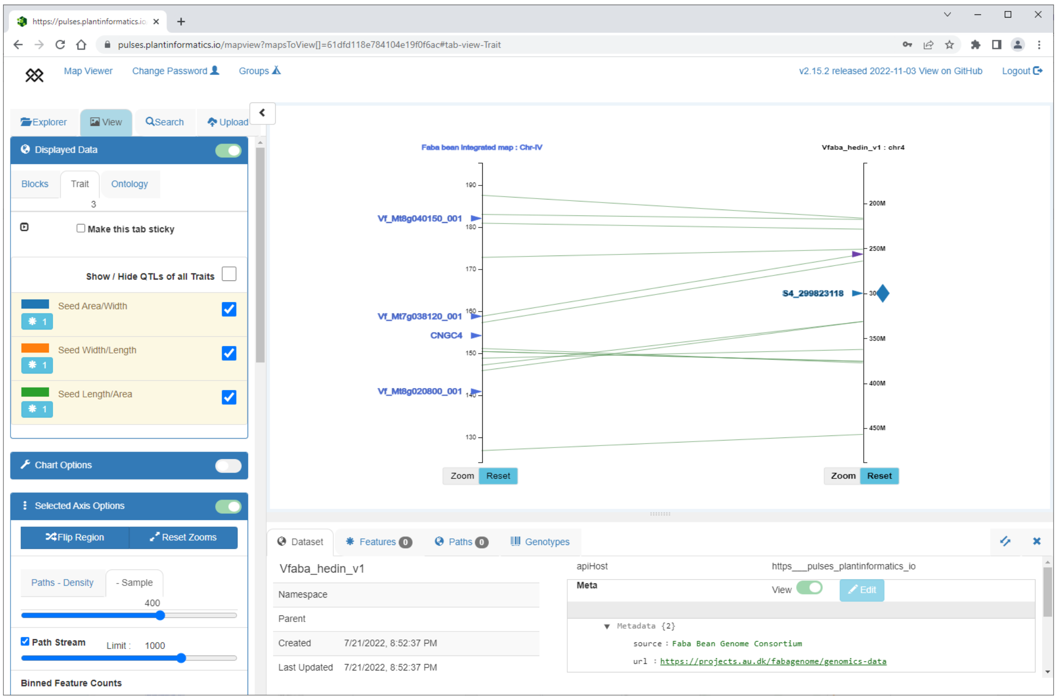

The resulting visualisation shows the S4_299823118 GWAS peak marker for Seed Area/Width as reported in this paper co-locates with the seed weight QTL reported in Khazaei et al. 2014.
