# Supplementary material for: The giant diploid faba genome unlocks variation in a global protein crop
Source: Nature. 2023 Mar 8;615(7953):652–9. doi: 10.1038/s41586-023-05791-5 (PMC10033403; doi:10.1038/s41586-023-05791-5)
Supplement: Supplementary file 2 — Reporting Summary [file 41586_2023_5791_MOESM2_ESM.pdf]

nature portfolio

Corresponding author(s): Donal M. O'Sullivan
Alan H. Schulman
Stig U. Andersen

Last updated by author(s): Jan 24, 2023

Reporting Summary

Nature Portfolio wishes to improve the reproducibility of the work that we publish. This form provides structure for consistency and transparency in reporting. For further information on Nature Portfolio policies, see our Editorial Policies and the Editorial Policy Checklist.

Statistics

For all statistical analyses, confirm that the following items are present in the figure legend, table legend, main text, or Methods section.

n/a	Confirmed	
☐	☒	The exact sample size (*n*) for each experimental group/condition, given as a discrete number and unit of measurement
☒	☐	A statement on whether measurements were taken from distinct samples or whether the same sample was measured repeatedly
☐	☒	The statistical test(s) used AND whether they are one- or two-sided<br>*Only common tests should be described solely by name; describe more complex techniques in the Methods section.*
☒	☐	A description of all covariates tested
☒	☐	A description of any assumptions or corrections, such as tests of normality and adjustment for multiple comparisons
☐	☒	A full description of the statistical parameters including central tendency (e.g. means) or other basic estimates (e.g. regression coefficient) AND variation (e.g. standard deviation) or associated estimates of uncertainty (e.g. confidence intervals)
☐	☒	For null hypothesis testing, the test statistic (e.g. *F*, *t*, *r*) with confidence intervals, effect sizes, degrees of freedom and *P* value noted<br>*Give P values as exact values whenever suitable.*
☒	☐	For Bayesian analysis, information on the choice of priors and Markov chain Monte Carlo settings
☒	☐	For hierarchical and complex designs, identification of the appropriate level for tests and full reporting of outcomes
☐	☒	Estimates of effect sizes (e.g. Cohen's *d*, Pearson's *r*), indicating how they were calculated

Our web collection on statistics for biologists contains articles on many of the points above.

Software and code

Policy information about availability of computer code

Data collection	No software was used for data collection.
Data analysis	Multiple published software packages were used in the analysis including: ADMIXTURE v1.3.0, Beagle v5, bedtools v2.30.0, BLASTP v2.2.26, BLAST v2.12.0, BLINK (no version), BlobTools v1.1, BRAKER v2.1.6, BUSCO v3.0.2b, BUSCO v5.2.2, cd-hit-est v4.8.1, CLC Genomics Workbench 11, Clustal Omega v1.2.4, CPC2 v2.0, CRBHits v0.0.4, cutadapt v1.15, DANTE v0.1.1, DupGen_finder v25Apr2019, EMMAX (no version), FarmCPU (no version), featureCounts, subread 2.0.1, findGSE v1.94, FlexiDot v1.06, GAPIT3 v3.1, GEMMA v0.98.5, GenomeScope v1.0, GMAP v2020-10-14, GSAlign v1.0.22, hifiasm v0.11-r302, hifiasm v0.15.5-r350, Jellyfish v2.2.10, KaKs_Calculator v1.2, Kallisto v 0.44.0, KAT v2.4.2, Kraken2 v2.1.1, LDBlockShow v1.40, Liftoff v1.6.1, LTRharvest (no version), LTR_retriever v2.9.0, MCMCTREE v4.4, MCScanX v2.0, MEGA X v10.2.6, Merqury v1.3,minimap2 v2.20, minimap2 v 2.24-r1122, Novosort v3.06.05, Orthofinder v2.5.4, OrthoMCL v2.0.9, PAML v4.5, PhyML v3.0, prot-scriber v0.1.0, purge_haplotigs v1.1.2, regioneR v1.18.1, RepeatMasker v4.1.1, RepeatMasker v4.2.1, RepeatModeler v2.0.1, RGAugury v1.0, rrBLUP v4.6.1, SAMtools v1.15.1, Sniffles v1.0.11, Sniffles v2.0.7, SNPEff v4.3, STAR 2.7.8a, TreeBest v1.9.2, TRITEX pipeline (no version)

For manuscripts utilizing custom algorithms or software that are central to the research but not yet described in published literature, software must be made available to editors and reviewers. We strongly encourage code deposition in a community repository (e.g. GitHub). See the Nature Portfolio guidelines for submitting code & software for further information.

Data

Policy information about availability of data

All manuscripts must include a data availability statement. This statement should provide the following information, where applicable:

- Accession codes, unique identifiers, or web links for publicly available datasets
- A description of any restrictions on data availability
- For clinical datasets or third party data, please ensure that the statement adheres to our policy

Raw data are available under European Nucleotide Archive (ENA) study ID PRJEB52541. Genome assemblies and annotations for Hedin/2 and Tiffany are available for download at www.fabagenome.dk and can be accessed via an Interactive Genome Browser (http://w3lamc.umbr.cas.cz/lamc/resources.html).
Publicly accessible expression data used: PRJNA395480, SRS8798224-SRS8798245
Databases used in the study: BUSCO databases: embryophyta_odb9, embryophyta_odb10, fabales_odb10 (https://busco.ezlab.org/), REXdb v3.0 (http://repeatexplorer.org/?page_id=918), satDNA (10.1093/molbev/msaa090); Viridiplantae OrthoDB v10.1 (https://www.orthodb.org/), RepBase (https://www.girinst.org/repbase/, release 20181926), GyDB v2.0 (https://gydb.org/index.php?title=Main_Page)

Human research participants

Policy information about studies involving human research participants and Sex and Gender in Research.

Reporting on sex and gender	N/A
Population characteristics	N/A
Recruitment	N/A
Ethics oversight	N/A

Note that full information on the approval of the study protocol must also be provided in the manuscript.

Field-specific reporting

Please select the one below that is the best fit for your research. If you are not sure, read the appropriate sections before making your selection.

☒ Life sciences ☐ Behavioural & social sciences ☐ Ecological, evolutionary & environmental sciences

For a reference copy of the document with all sections, see nature.com/documents/nr-reporting-summary-flat.pdf

Life sciences study design

All studies must disclose on these points even when the disclosure is negative.

Sample size	The two accessions chosen for genome sequencing were selected based on their importance for the faba bean community. Hedin/2 was used for the development of genomic resources. Tiffany is a modern elite cultivar. They also have a contrasting hilum colour phenotype. Accessions for SPET genotyping were chosen to represent world-wide diversity. No sample size calculation was performed.
Data exclusions	No data was excluded.
Replication	Transcriptomics experiments (profiling of seed, root and nodule gene expression) were performed in triplicates. Field trials were performed across two locations: trials at Sejet Plant Breeding, Sejet (55.82°N, 9.94°E) in 2019 (trial 23), 2020 (trial 26), and 2021 (trial 30) and at Nordic Seed, Dyngby (55.96°N, 10.25°E) in 2018 (trial 11), 2019 (trial 22) and 2020 (trial 25). All attempts at replication were successful and replicates were used in the study. Where applicable, number of replicates is indicated in the methods and supplementary material.
Randomization	Randomization does not directly apply to genome sequencing and assembly studies. In cases where randomization procedures are part of computational analyses (for example bootstrapping used in phylogenetic inference) existing community standards were used.
Blinding	Study focuses on plant genome assembly and genomic analyses. The study design did not require and involve blinding.

Reporting for specific materials, systems and methods

We require information from authors about some types of materials, experimental systems and methods used in many studies. Here, indicate whether each material, system or method listed is relevant to your study. If you are not sure if a list item applies to your research, read the appropriate section before selecting a response.

Materials & experimental systems

n/a	Involved in the study
☒	☐ Antibodies
☒	☐ Eukaryotic cell lines
☒	☐ Palaeontology and archaeology
☒	☐ Animals and other organisms
☒	☐ Clinical data
☒	☐ Dual use research of concern

Methods

n/a	Involved in the study
☐	☒ ChIP-seq
☐	☒ Flow cytometry
☒	☐ MRI-based neuroimaging

ChIP-seq

Data deposition

☒ Confirm that both raw and final processed data have been deposited in a public database such as GEO.

☐ Confirm that you have deposited or provided access to graph files (e.g. BED files) for the called peaks.

Data access links
May remain private before publication.

For "Initial submission" or "Revised version" documents, provide reviewer access links. For your "Final submission" document, provide a link to the deposited data.

Files in database submission

Provide a list of all files available in the database submission.

Genome browser session
(e.g. UCSC)

Provide a link to an anonymized genome browser session for "Initial submission" and "Revised version" documents only, to enable peer review. Write "no longer applicable" for "Final submission" documents.

Methodology

Replicates

Describe the experimental replicates, specifying number, type and replicate agreement.

Sequencing depth

Describe the sequencing depth for each experiment, providing the total number of reads, uniquely mapped reads, length of reads and whether they were paired- or single-end.

Antibodies

Describe the antibodies used for the ChIP-seq experiments; as applicable, provide supplier name, catalog number, clone name, and lot number.

Peak calling parameters

Specify the command line program and parameters used for read mapping and peak calling, including the ChIP, control and index files used.

Data quality

Describe the methods used to ensure data quality in full detail, including how many peaks are at FDR 5% and above 5-fold enrichment.

Software

The centromere regions were identified in each chromosome using ChIP-seq with the CENH3 (a centromere-specific histone H3 variant) antibody reported by Avila Robledillo Let al. Briefly, the raw reads from the ChiP-seq were trimmed by cutadapt (v.1.15) and mapped to the preliminary pseudomolecules using minimap2. The alignments were converted to BAM format using SAMtools and sorted by Novosort (V3.06.05) (http://www.novocraft.com). The read depth was then calculated in 100 kb windows.

Flow Cytometry

Plots

Confirm that:

☒ The axis labels state the marker and fluorochrome used (e.g. CD4-FITC).

☒ The axis scales are clearly visible. Include numbers along axes only for bottom left plot of group (a 'group' is an analysis of identical markers).

☐ All plots are contour plots with outliers or pseudocolor plots.

☐ A numerical value for number of cells or percentage (with statistics) is provided.

Methodology

Sample preparation

Nuclear genome size was estimated by flow cytometry as described previously (Doležel et al., 2007). Briefly, intact leaf tissues of a V. faba accession Hedin/2 and Secale cereale cv. Dankovske (2C = 16.19 pg DNA; Doležel et al. 1992), which served as the internal reference standard, were chopped together in a glass Petri dish containing 500 µl Otto I solution (0.1M citric acid, 0.5% v/v Tween 20; Otto, 1990). The crude suspension was filtered through a 50 µm nylon mesh. Nuclei were then pelleted (300 × g, 2 min) and resuspended in 300 µl of Otto I solution. After 15 min of incubation on ice, 600 µl of Otto II solution supplemented with 50 µg/ml RNase and 50 µg/ml propidium iodide were added.

Instrument

Samples were analyzed using a CyFlow Space flow cytometer (Sysmex Partec GmbH, Görlitz, Germany) equipped with a 532

Instrument	nm green laser. The gain of the instrument was adjusted so that the peak representing G1 nuclei of the standard was positioned approximately on channel 100 on a histogram of relative fluorescence intensity when using a 512-channel scale
Software	Analysis was performed using FloMax software (Sysmex Partec GmbH, Görlitz, Germany) and 2C DNA contents (in pg) were calculated from the means of the G1 peak positions by applying the formula: 2C nuclear DNA content = (sample G1 peak mean) × (standard 2C DNA content) / (standard G1 peak mean). The mean nuclear DNA content (2C) was then calculated for each species. DNA contents in pg were converted to genome size in bp using the conversion factor 1 pg DNA = 0.978 Gbp (Doležel et al., 2003).
Cell population abundance	12 individual Hedin/2 plants were sampled, and each sample was analyzed three times, each time on a different day. A minimum of 5000 nuclei per sample were analyzed.
Gating strategy	The low level threshold was set to channel 20 to eliminate particles with the lowest fluorescent intensity from the histogram, all remaining fluorescent events were recorded with no further gating used

☐ Tick this box to confirm that a figure exemplifying the gating strategy is provided in the Supplementary Information.
